# Efficacy of *Acacia nilotica, Eucalyptus camaldulensis,* and *Salix safsafs* on the mortality and development of two vector-borne mosquito species, *Culex pipiens and Aedes aegypti*, in the laboratory and field

**DOI:** 10.1016/j.heliyon.2023.e16378

**Published:** 2023-05-19

**Authors:** Rowida S. Baeshen, Mohamed M. Baz

**Affiliations:** a Department of Biology, Faculty of Science, University of Tabuk, Tabuk, 71421, Saudi Arabia; b Department of Entomology, Faculty of Science, Benha University, Benha 13518, Egypt

**Keywords:** *Acacia nilotica*, *Aedes aegypti*, *Culex pipiens*, Medicinal plants, Phytochemicals, Photosensitizer

## Abstract

Mosquitoes are one of the most lethal animals in the world and transmit many dangerous human pathogens, causing millions of deaths each year. The search for modern and better mosquito control is an endless effort almost all over the world. Phytochemicals are promising biological agents for getting rid of pests that are harmful to human and animal health and crops, they are inexpensive, biodegradable, and have diverse modes of action. The efficacy of acetone and hexane leaf extracts of *Acacia nilotica, Eucalyptus camaldulensis,* and *Salix safsafs* was investigated against the 2nd and 4th larvae and pupae of two vectors, *Culex pipiens* and *Aedes aegypti*. The results showed the obvious effect *of A. nilotica* extract on the mortality of mosquito larvae, the reduction of female eggs, and a higher mortality rate in sunlight than in shadow (fluorescein). Data from field trials revealed that *A. nilotica* extracts had the greatest effect on larval reduction, reaching 89.8% in 24 h and having a 12-day stability. Polyethylene glycol, sesquiterpenes, and fatty acids were the most common compounds found in *A. nilotica, E. camaldulensis,* and *S. safsafs*, respectively. The acacia plant had promising larvicidal activity, safe and effective alternative to chemical insecticides.

## 1 Introduction

Plants contain phytochemicals, or secondary metabolics, and ingesting them typically has positive health impacts. Due to their positive impacts on human health and significant health benefits for consumers, phytochemicals are of great interest and have significant antioxidant potential [[1], [2], [3]]. According to preclinical, clinical, and epidemiological research, phytochemicals may be useful in treating a variety of ailments because of their antioxidant and anti-inflammatory properties. On the other hand, consuming some phytochemicals may have some short-term and long-term harmful effects, including the potential to cause cancer and many other diseases [4].

Aromatic medicinal plants have been used to treat many diseases in humans and animals, both in the medical field and in industry. As a result, consumers who care about the environment are using more natural insecticides in many parts of their lives [5,6].

Besides that, increased demand for food to feed the ever-growing population and the spreading of insect diseases led to the development and adoption of biopesticides as a quick and effective strategy for controlling crop pests. However, botanical pesticides have not been fully adopted due to challenges in formulation and commercialization, which are attributed to a lack of chemical data and positive controls [[6], [7], [8]].

Mosquitoes are one of the most important groups of insects that are known for their public health importance in transmitting many dangerous diseases to humans and animals. In addition to being irritating and hypersensitive to their stings for everybody, they also transmit deadly diseases such as filariasis, malaria, dengue fever, yellow fever, and Japanese encephalitis. These results in more than a million victims a year worldwide, especially in tropical and subtropical countries, where poverty and social decay contribute to the spread of diseases [9,10].

Synthetic insecticides are still considered one of the most basic and effective means of controlling the transmission of these mosquito-borne diseases. Because of their rapid action, synthetic insecticides are the first line of defense, but continued use of synthetic insecticides may lead to the development of resistance and a permanent residual effect on the bio-environment, which may be harmful to humans and include non-targeted organisms. When applied carelessly, they may also cause unwanted, severe, and long-term side effects. Therefore, their frequent and continuous use led to the emergence and development of insect and mosquito resistance to industrial pesticides without eliminating the ongoing risks of epidemics [11]. To reduce this, it is important to look for alternative insecticides that are easily biodegradable.

Environmentally friendly and non-toxic bio-sourced products are regaining appeal in many aspects of our lives as a result of the public's increased awareness of environmental health and safety issues. Researchers are seeking to find safe alternatives to synthetic pesticides, such as plant pesticides, as they can be easily degraded. As they are largely ecologically safe, biodegradable, environmentally friendly, target-specific, and non-toxic to non-target animals like people, plant insecticides may one day serve as viable replacements for synthetic pesticides [12,13].

Phytochemistry is perhaps one of the oldest areas of research in science, but despite excellent and renewed chemical exploration, new insights into medicinal and other plants are still needed to better understand their intrinsic complexity and exploit their enormous commercial potential, along with their broad applications. Many unique and renewable natural phytochemical compounds are discovered in the medical, commercial, and industrial fields that are concerned with aromatic medicinal plants. Currently, plant extracts and other biomaterials are being studied in an effort to reduce the number of mosquitoes and other insect vectors. Researchers have discovered that the plant contains antioxidant, antimalarial, anticancer, antiplasmodial, antimolluscicidal, antifungal, antimicrobial, as well as HCV and HIV–I inhibitory action [14,15].

Green plants are a natural source for many different products that we use on a daily basis, and the main driver in this is photosynthesis, which plays a crucial role in the production of insect growth regulators, insect pheromones, photoinsecticides and insect repellents, which in turn affect the effectiveness and mechanism of natural pesticides. Photosensitizers are non-toxic to humans, but they are extremely toxic to pests. So, they can master a tool to control pests, including natural plant extracts such as curcumin, rose bengal, gamboge tree resin, garcinia, mangosteen peel, etc. They have shown great potential as a photosensitizer (light-sensitive) [[16], [17], [18]].

Natural dyes, which are obtained from plants, insects, and minerals and are biologically sourced, are renewable, sustainable products with minimal environmental impact and have been known since ancient times for their use in coloring textiles, cosmetics, and food ingredients. With photodynamic therapy (photosensitizers), these natural dyes have been used in numerous scientific and industrial applications, such as clean energy production [19,20]; dyeing [21]; printing [22]; water purification [23]; insecticides for photosensitizer manufacturing, etc. [[24], [25], [26]].

*Acacia nilotica* is among the approximately 135 species of thorny African acacias that are naturally widespread in drier regions, both in Africa and Asia. The species is well known by its regional names, "Sunt" for the tree and "Garad" for the fruit pods, and the tree grows natively in deep forests, primarily next to rivers, streams, and rain catchments. It has long been used in Asia for various traditional purposes, such as treating cancer, colds, coughs, fevers, tumors, hemorrhages, typhoid, convalescence, intestinal pain, and diarrhea. The tree's bark, gum, leaves, and pods are also widely used in Africa to treat many ailments [27,28]. *Acacia nilotica* seeds and leaves have shown promise as fungicides, bactericides, molluscicides, parasiticides and insecticides [27]. *Acacia nilotica*, also known as *Vachellia nilotica*, is a member of the Fabaceae family and is located in Egypt, India and other countries. Both humans and animals can be treated with the help of this herb.

Phytochemistry have shown that the stem bark of *A. nilotica* has terpenoids, alkaloids, saponins, cardiac glycosides, and tannins [29]. These secondary metabolites are crucial in the interactions between plants and insects and are what give plants their insect resistance [30].

*Eucalyptus camaldulensis* is an evergreen plant in Egypt and is widely cultivated throughout the country and a large part of the world, especially around fresh water. This genus, originally from Australia (Myrtaceae), includes more than 700 species, and the *Eucalyptus* tree is one of the most diverse among flowering trees with multiple stems [31,32]. Most of these species have been widely used in traditional and modern medicine to treat many diseases, such as lung diseases and coughing treated with medicines such as expectorants [33]. It also has anti-tuberculosis, antibacterial, antifungal, and microbiological antiseptic properties [34,35], and the cineole in its leaves kills mosquito larvae and keeps adults away [36,37].

*Salix safsaf*, the deciduous herb, cape silver willow, or safsaf willow, is another name for the little tree known as the Egyptian willow, which has been found in Egypt since prehistoric times [38,39]. Typically, it can be found in moist places like those near waterways and on the Nile River in Egypt. Salicin willow, another name for white willow, has long been used for its therapeutic properties [40]. Its branches are supple, long, and thin. Over many generations, the plant's leaves, seeds, and other parts have been used in traditional medicine to relieve inflammation, pain, and fever [41].

Phytochemicals still contain many natural, organic compounds that have a toxic or repellent effect on many insect pests, which is what we aim to achieve and explore through this work. Therefore, the trees chosen are available in the Egyptian environment, in addition to the fact that it contains various toxic substances. The *Acacia* tree needs a lot of attention, whereas this tree has been used since the Pharaonic time because it (*Acacia* or Sunt) contains substances with unique properties [42]. These secondary metabolites are crucial in the interactions between plants and insects and are what give plants their insect resistance [30].

**Fig. 1:**
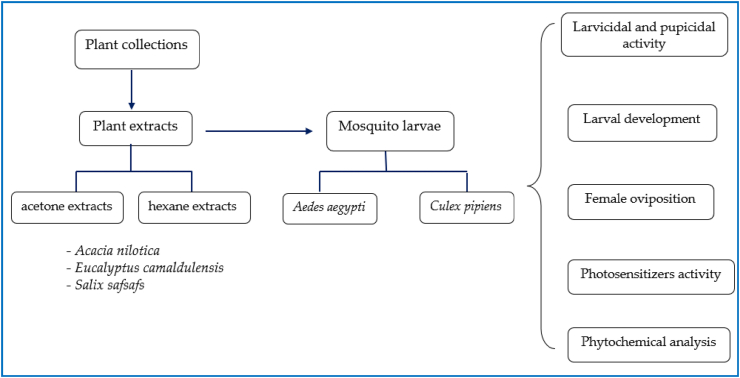
Flowchart of research structure.

This study aimed to evaluate the larvicidal effectiveness of plant leaf extracts, *Acacia nilotica, Eucalyptus camaldulensis,* and *Salix safsafs* against 2nd, 4th larvae and pupae of *Culex pipiens* and *Aedes aegypti* besides determining some biological aspects after being treated with sub-lethal concentrations of plant extracts. The structure chart of the article is shown in Fig. 1.

## 2 Materials and methods

### 2.1 Mosquito colony

Mosquito larvae, *Aedes aegypti* and *Culex pipiens* used for all investigations were obtained from Department of Entomology, Faculty of Science, Benha University. Mosquito larvae were reared in enamel plates (25 × 20 × 10 cm) filled with 2 L de-chlorinated tap water and given Tetramin® fish food and powdered dog biscuits every 2 day. At 27 ± 2 °C, 75–80% RH, and a 12:12 h (L/D) photoperiod, the colony was kept in good condition. After pupation, *Ae. aegypti* was transferred into mosquito cage (30 × 25 × 25 cm) and *Cx. pipiens* pupae into (30 × 30 × 30 cm) cages in an insectary. Mosquito adults used 8–10% sucrose solution as food. Egg-laying dishes for *Ae. aegypti* mosquitoes are provided with a paper or a thin foam layer that sticks to the edge of the egg-laying dish to help females stand on it while laying eggs, in addition to facilitating the collection of eggs through that sheet. Both larval species were kept in the identical laboratory settings and were continually available for the tests [43].

### 2.2 Collection of plant materials

**Fig. 2:**
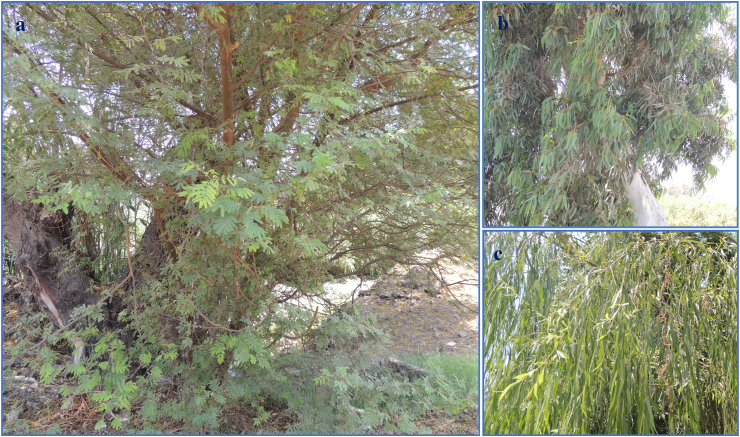
Tree of *Acacia nilotica* (a), *Eucalyptus camaldulensis* (b), and *Salix safsaf* (c).

Leaves of *Acacia nilotica* (Fabaceae), *Eucalyptus camaldulensis* (Myrtaceae), and *Salix safsafs* (Salicaceae) were collected from different areas in Egyptian villages, Giza area, Egypt in May–September 2021 (Fig. 2). Plants were identified at Flora and Phytotaxonomic section, Agricultural Research Center, Giza, Egypt.

### 2.3 Preparation of plant extracts

Fresh leaves of *A. nilotica, E. camaldulensis,* and *S. safsafs* were washed with tap water and air dried. Stock solutions of plant leaves were extracted by mechanically grinding 50 g using a stainless-steel electric mixer and placing the powder in a Soxhlet apparatus for 4–7 h according to the type of solvent (acetone and hexane). The solution was filtered using Whatman No. 1 filter paper and dried in an oven at 30 °C for 6 h. The extracts were stored in a dark bottle in a refrigerator at −5 °C for 24 h prior to the experiment.

### 2.4 Larvicidal and pupicidal efficacy

Plant extracts were tested for their larvicidal and pupicidal efficacy according to WHO [44]. against 2nd, 4th larval instar and pupal stage of *Cx. pipiens* and *Ae. aegypti*. Different concentrations were prepared as 62.5, 125, 250, 500, 1000, and 1500 ppm with twenty larvae were placed in a glass beaker containing 250 ml for two species. The experiment as well as the control group were treated with the solvent only. Five replicates were used for each concentration. *Cx. pipiens* and *Ae. aegypti* larval and pupal mortalities were recorded after 24 and 48 h post-treatment (PT).

### 2.5 Effect of plant extracts on larval development

The effect of sublethal (LC_50_: lethal concentrations of cumulative effect) of the plant extracts, *A. nilotica, E. camaldulensis,* and *S. safsafs* was tested through LC_50_ values which were determined with the test larvicide after 48 h post-treatment with plant extracts. There were five replicates, each containing a total of 30 *Cx. pipiens* and *Ae. aegypti* from the 3rd larval instar. Daily readings were performed to check for larval stage, behavioral changes, the presence of exuvia, the emergence of adults, and potential mortality of larvae, pupae, and adults. The experiment was conducted until the death of the last pupae, or the last adult completely emerged at room temperature (27 ± 2 °C).

### 2.6 Effect of plant extracts on egg laying and hatching percentages

Testing of the efficacy of plant extracts on female egg-laying activity was performed using 20 females (3–5 days old) from both *Cx. pipiens* and *Ae. aegypti* after treating their larvae with their LC_50_ and LC_90_ values and monitoring egg laying in a bioassay cage (30 cm × 30 cm x 30 cm) containing cups with 350 ml of dis-tilled water for egg laying. Adults were given a steady supply of an 8% glucose solution. A piece of filter paper (Whatman® No. 1) was placed on the inner surface of each plastic cup to act as a support for female *Ae. aegypti* eggs. Under a stereomicroscope, the number of eggs laid in the cups for each treatment group was recorded.

### 2.7 Larvicidal activity of plant extracts as photosensitizer

The effect of plant extracts (*A. nilotica, E. camaldulensis, and S. safsafs*) was tested against *Cx. pipiens* and *Ae. aegypti* through a larval dipping test, according to a previously described protocol [44], after 24 h of post-treatment to determine the efficacy of plant extracts as photosensitizers. Two concentrations of plant extracts (62.5 and 125 ppm) were freshly prepared in distilled water. There were five replicates, each containing a total of 100 *Cx. pipiens* and *Ae. aegypti* from the 4th larval instar.

### 2.8 Phytochemicals analysis

Thermo Scientific Trace GC Ultra/ISQ Single Quadrupole MS, TG-5MS fused silica capillary column, 0.1 mm, 0.251 mm, and 30 m thick were utilized for the GC/MS was employed for the biochemical analyses. It was achieved using an electronic ionizer with a 70 eV ionization energy. As a carrier gas, helium gas was used (flow rate: 1 ml/min). The MS transmission line and injector were both set to 280 °C. The oven was preheated to 50 °C, then increased to 150 °C at a rate of 7 °C per minute, 270 °C at a rate of 5 °C per minute (pause for 2 min), and lastly 310 °C at a rate of 3.5 °C per min (continued for 10 min). A relative peak area was employed to explore the quantification of all components discovered. The chemicals were at least partially identified by comparing the retention times and mass spectra of the chemicals to those of NIST, Willy Library data from the GC/MS instrument. Identification was done using the aggregate spectrum of user-generated reference libraries. To evaluate peak homogeneity, single-ion chromatographic reconstructions were performed. To verify GC retention periods, co-chromatographic analysis of reference substances was used whenever practical [45].

### 2.9 Larvicidal field assessment

In Kafr Saad village, Qalyubiya Governorate, Egypt, pools were selected (with average lengths of 6.5 m, 2.0 m, and 0.45 m) for the assessment of plant extracts. The pools used for larval breeding were chosen because they had a high density of mosquito stages. Each plant extract was applied in the breeding areas with a dose of LC95 × 2. Three replicates of each treatment were carried out. mosquito larvae were collected in field water from each site using an enamel pad (450 mL) in each larvicidal treatment [46].

### 2.10 Data analyses

The data were analyzed by the software, SPSS V23 (IBM, USA), for doing the Probit analyses to calculate the lethal concentration (LC) values and the one-way analysis of variance (ANOVA) (Post Hoc/Turkey's HSD test). The significant levels were set at p < 0.05.

## 3 Results

### 3.1 Larvicidal activity

This work evaluated three plant extracts of *Acacia nilotica, Eucalyptus camaldulensis,* and *Salix safsafs* against the 2nd, 4th larval instars, and pupae of *Culex pipiens* and *Aedes aegypti*, and expressed dose and time-dependent efficacy extended at 2 days post-treatment.

The mortality % 24 h post-treatment using 1500 ppm, acetone extracts of *Acacia nilotica, Eucalyptus camaldulensis,* and *Salix safsafs* were 100% for 2nd, 100, 95, and 100% for 4th larval instar and 82, 70, and 77% for pupae of *Cx. pipiens* with LC_50_ (50%, median lethal concentration) = 152.16, 267.77, 207.32 ppm, 175.71, 374.15, 357.30 ppm, and 429.64, 686.81, and 523.39 ppm, respectively); whereas those of hexane extracts were 100% for 2nd and 4th larval instars and 78, 65, and 74%, for pupal stage, respectively post-treatments with 1500 ppm (LC_50_ = 178.75, 216.4, 248.39 ppm, 249.30, 341.29, 357.3 ppm, and 567.42, 834.88, and 597.8 ppm, for 2nd, 4th larval instars, and pupal stage respectively) (Table 1, Table 2).

**Table 1:** Efficacy of *Acacia nilotica, Eucalyptus camaldulensis, and Salix safsafs* on *Culex pipiens*, 24 h post-treatment.

Plant extract	Conc. (ppm)	Acetone			Hexane		
		2nd	4th	Pupae	2nd	4th	Pupae
*Acacia nilotica*	0	00.0±0^fA^	00.0±0^fA^	00.0±0^gA^	00.0±0^fA^	00.0±0^gA^	00.0±0^gA^
	62.5	18 ± 2.55^eA^	14 ± 1.87^eB^	9 ± 2.92^fC^	15 ± 2.24^eA^	8 ± 2.00^fB^	5 ± 2.24^fC^
	125	37 ± 2.00^dA^	33 ± 3.39^dB^	18 ± 3.39^eC^	31 ± 4.00^dA^	20 ± 2.24^eB^	14 ± 1.87^eC^
	250	70 ± 2.24^cA^	65 ± 3.54^cB^	36 ± 2.45^dC^	62 ± 3.00^cA^	49 ± 6.78^dB^	32 ± 1.22^dC^
	500	92 ± 2.55^bA^	85 ± 3.54^bB^	57 ± 4.06^cC^	86 ± 1.87^bA^	76 ± 1.87^cB^	53 ± 4.64^cC^
	1000	100 ± 0.00^aA^	100 ± 0.00^aA^	69 ± 4.00^bB^	100 ± 0.00^aA^	95 ± 3.16^bB^	65 ± 3.54^bC^
	1500	100 ± 0.00^aA^	100 ± 0.00^aA^	82 ± 3.39^aB^	100 ± 0.00^aA^	100 ± 0.00^aA^	78 ± 2.55^aB^
*Eucalyptus camaldulensis*	0	00.0±0^gA^	00.0±0^gA^	00.0±0^fA^	00.0±0^gA^	00.0±0^gA^	00.0±0^fA^
	62.5	9 ± 1.87^fA^	7 ± 1.22^fB^	1 ± 1.00^fC^	12 ± 1.22^fA^	7 ± 1.22^fB^	00.0±0^fC^
	125	20 ± 1.58^eA^	15 ± 2.24^eB^	12 ± 2.55^eC^	25 ± 1.58^eA^	16 ± 1.87^eB^	7 ± 2.55^eC^
	250	41 ± 4.30^dA^	34 ± 2.92^dB^	27 ± 2.55^dC^	58 ± 3.39^dA^	40 ± 4.47^dB^	22 ± 3.00^dC^
	500	70 ± 3.54^cA^	58 ± 3.74^cB^	45 ± 3.54^cC^	77 ± 1.22^cA^	65 ± 3.54^cB^	40 ± 5.7^cC^
	1000	95 ± 2.74^bA^	78 ± 6.04^bB^	57 ± 2.55^bC^	96 ± 2.45^bA^	86 ± 2.92^bB^	52 ± 2.55^bC^
	1500	100 ± 0.00^aA^	95 ± 2.24^aB^	70 ± 3.16^aC^	100 ± 0.00^aA^	100 ± 0.00^aA^	65 ± 4.18^aB^
*Salix safsafs*	0	00.0±0^gA^	00.0±0^gA^	00.0±0^gA^	00.0±0^gA^	00.0±0^gA^	00.0±0^gA^
	62.5	14 ± 1.87^fA^	9 ± 1.87^fB^	5 ± 2.24^fC^	11 ± 1.87^fA^	5 ± 1.58^fB^	2 ± 1.22^fC^
	125	25 ± 2.24^eA^	21 ± 3.32^eB^	15 ± 2.24^eC^	20 ± 1.58^eA^	13 ± 1.22^eB^	12 ± 4.06^eC^
	250	55 ± 4.18^dA^	48 ± 3.39^dB^	32 ± 2.00^dC^	52 ± 2.00^dA^	35 ± 3.54^dB^	29 ± 2.92^dC^
	500	80 ± 3.16^cA^	71 ± 3.32^cB^	52 ± 4.06^cC^	74 ± 3.67^cA^	59 ± 5.79^cB^	49 ± 3.67^cC^
	1000	98 ± 1.22^bA^	90 ± 3.54^bB^	65 ± 3.16^bC^	95 ± 3.16^bA^	82 ± 3.74^bB^	62 ± 3.74^bC^
	1500	100 ± 0.00^aA^	100 ± 0.00^aA^	77 ± 2.55^aB^	100 ± 0.00^aA^	100 ± 0.00^aA^	74 ± 4.30^aB^

a, b & c …. etc: There is no significant difference (p > 0.05) between any two means, within the same column have the same superscript letter; A, B & C … etc: There is no significant difference (p > 0.05) between any two means for the same solvent, within the same row have the same superscript letter. Five replicates were used for each concentration (20 larvae/replicate were used).

**Table 2:** Lethal concentrations of *Acacia nilotica, Eucalyptus camaldulensis,* and *Salix safsafs* against *Culex pipiens*, 24 h post-treatment.

Stage	Plant extract	Solvents	LC_50_ (95% CL) *	LC_90_ (95% CL)	LC_95_ (95% CL)	Equation**	X^2^
2nd	*Acacia nilotica*	Acetone	152.1 (134.4–170.9)	445.7 (380.8–541.8)	604.5 (501.7–766.4)	2.745 ± 0.201	3.11
		Hexane	178.7 (158.2–200.7)	539.0 (460.0–655.0)	737.1 (611.2–932.9)	2.673 ± 0.186	5.54
	*Eucalyptus camaldulensis*	Acetone	267.7 (237.7–300.7)	850.7 (722.4–1038.0)	1180.6 (974.0–1499.0)	2.552 ± 0.166	9.20
		Hexane	216.4 (191.2–243.1)	733.7 (630.2–878.7)	1037.1 (867.0–1289.0)	2.146 ± 0.142	2.95
	*Salix safsafs*	Acetone	207.3 (183.2–233.3)	662.0 (562.2–808.2)	920.0 (758.6–1170.6)	2.541 ± 0.171	7.14
		Hexane	248.3 (218.2–281.8)	878.0 (728.8–1107.3)	1255.8 (1006.7–1663.1)	2.337 ± 0.164	8.20
4th	*Acacia nilotica*	Acetone	175.7 (155.4–197.3)	529.9 (452.1–644.1)	724.5 (600.6–917.5)	2.673 ± 0.187	4.69
		Hexane	249.3 (222.2–278.4)	770.8 (665.4–917.8)	1061.5 (893.7–1307.8)	2.614 ± 0.157	2.21
	*Eucalyptus camaldulensis*	Acetone	374.1 (328.3–426.4)	1503.5 (1224.2–1943.7)	2230.30 (1746.6–3041.5)	2.121 ± 0.145	5.06
		Hexane	341.2 (296.4–393.3)	1448.0 (1149.4–1949.8)	2181.2(1654.8–3130.7)	2.041 ± 0.155	2.39
	*Salix safsafs*	Acetone	261.8 (231.1–295.5)	902.9 (759.3–1115.7)	1282.4 (1045.0–1655.4)	2.383 ± 0.157	5.44
		Hexane	357.3 (317.6–401.3)	1190.5 (1005.3–1462.2)	1674.6 (1372.3–2142.3)	2.451 ± 0.155	8.99
Pupae	*Acacia nilotica*	Acetone	429.6 (366.2–507.1)	2682.0 (1981.2–4002.7)	4507.5 (3127.8–7349.3)	1.611 ± 0.128	1.07
		Hexane	567.4 (486.1–671.5)	3354.5 (2450.1–5101.0)	5551.2 (3809.3–9220.3)	1.660 ± 1.33	2.21
	*Eucalyptus camaldulensis*	Acetone	686.8 (583.1–826.3)	4027.9 (2874.8–6349.5)	6650.6 (4451.6–11488.8)	1.668 ± 0.138	4.82
		Hexane	834.8 (707.8–1012.3)	4502.9 (3197.4–7179.6)	7260.5 (4871.7–12668.3)	1.751 ± 0.149	5.34
	*Salix safsafs*	Acetone	523.3 (446.7–620.4)	3135.4 (2295.2–4745.3)	5208.3 (3583.3–8605.3)	1.618 ± 0.132	1.39
		Hexane	597.8 (512.4–707.5)	3264.0 (2409.0–4882.8)	5281.2 (3676.4–8579.4)	1.738 ± 0.138	3.38

* LC50 values = lethal concentration that kills 50% of the exposed larvae; (95%CL) = lower and upper confidence limit; ** Regression line equation; X2 = chi-square; Significant at P < 0.05 level.

**Fig. 3:**
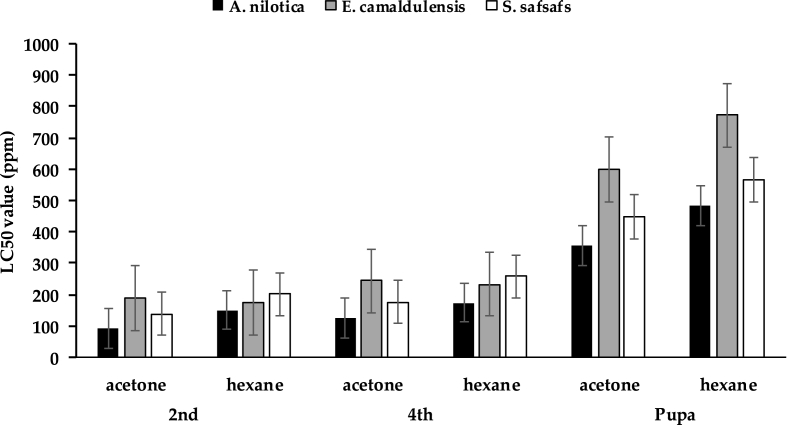
The mean number of larval mortalities induced by the effects of *Acacia nilotica, Eucalyptus camaldulensis, and Salix safsafs against* 3rd larval instars *Culex pipiens*, 48 h post-exposure.

Results showed that *A. nilotica* was the most effective regarding lethal concentrations (LC_50_ and LC_90_) and had lower values compared to other plant extracts either in acetone or hexane extracts for *Cx. pipiens*. Whereas at 48 h post-treatment, the LC_50_ values were 90.74, 123.31, and 357.85 ppm for 2nd, 4th larval instars, and pupal stage with acetone extracts, respectively (Fig. 3). While at hexane extracts, the LC_50_ values were 151.05, 174.44, and 481.7 ppm, respectively.

Unexpected, the morality recorded in *Eucalyptus camaldulensis* hexane extract was effective than acetone, where the morality reached 100, 96, and 54% for 2nd, 4th larval instars, and pupal stage respectively. In acetone extract, the mortality reached 100, 90, and 60% at 1000 ppm, 48 h post-treatment (Table 3).

**Table 3:** Efficacy of *Acacia nilotica, Eucalyptus camaldulensis,* and *Salix safsafs* on *Culex pipiens*, 48 h post-treatment.

Plant extract	Conc. (ppm)	Acetone			Hexane		
		2nd	4th	Pupae	2nd	4th	Pupae
*Acacia nilotica*	0	00.0±0^eA^	00.0±0^fA^	00.0±0^gA^	00.0±0^fA^	00.0±0^fA^	00.0±0^gA^
	62.5	26 ± 2.45^dA^	20 ± 2.24^eB^	12 ± 1.22^fC^	18 ± 2.00^eA^	14 ± 2.92^eB^	6 ± 1.87^fC^
	125	56 ± 2.92^cA^	48 ± 4.64^dB^	22 ± 4.06^eC^	38 ± 3.00^dA^	34 ± 3.32^dB^	15 ± 2.24^eC^
	250	92 ± 5.15^bA^	82 ± 2.55^cB^	40 ± 2.24^dC^	70 ± 2.74^cA^	64 ± 4.85^cB^	33 ± 2.00^dC^
	500	100 ± 0.00^aA^	98 ± 2.00^bB^	61 ± 2.92^cC^	92 ± 1.22^bA^	86 ± 1.87^bB^	55 ± 4.18^cC^
	1000	100 ± 0.00^aA^	100 ± 0.00^aA^	73 ± 2.55^bB^	100 ± 0.00^aA^	100 ± 0.00^aA^	67 ± 2.55^bB^
	1500	100 ± 0.00^aA^	100 ± 0.00^aA^	86 ± 4.30^aB^	100 ± 0.00^aA^	100 ± 0.00^aA^	80 ± 2.74^aB^
*Eucalyptus camaldulensis*	0	00.0±0^fA^	00.0±0^gA^	00.0±0^gA^	00.0±0^fA^	00.0±0^gA^	00.0±0^fA^
	62.5	15 ± 2.24^eA^	11 ± 2.92^fB^	3 ± 2.00^fC^	15 ± 1.58^eA^	12 ± 2.00^fB^	00.0±0^fC^
	125	34 ± 4.30^dA^	28 ± 4.64^eB^	15 ± 3.16^eC^	35 ± 3.54^dA^	25 ± 3.54^eB^	8 ± 2.00^eC^
	250	56 ± 4.00^cA^	48 ± 5.39^dB^	30 ± 3.54^dC^	62 ± 3.39^cA^	56 ± 5.10^dB^	22 ± 3.00^dC^
	500	81 ± 2.92^bA^	71 ± 5.57^cB^	48 ± 3.39^cC^	84 ± 2.92^bA^	75 ± 3.54^cB^	41 ± 4.85^cC^
	1000	100 ± 0.00^aA^	90 ± 2.74^bB^	60 ± 2.24^bC^	100 ± 0.00^aA^	96 ± 2.45^bB^	54 ± 1.87^bC^
	1500	100 ± 0.00^aA^	100 ± 0.00^aA^	74 ± 3.32^aB^	100 ± 0.00^aA^	100 ± 0.00^aA^	69 ± 2.92^aB^
*Salix safsafs*	0	00.0±0^fA^	00.0±0^gA^	00.0±0^gA^	00.0±0^gA^	00.0±0^gA^	00.0±0^gA^
	62.5	22 ± 5.15^eA^	16 ± 2.92^fB^	8 ± 1.22^fC^	13 ± 1.22^fA^	9 ± 1.87^fB^	3 ± 1.22^fC^
	125	42 ± 7.18^dA^	35 ± 4.47^eB^	18 ± 3.39^eC^	28 ± 1.22^eA^	21 ± 1.87^eB^	13 ± 3.39^eC^
	250	75 ± 7.42^cA^	64 ± 5.79^dB^	35 ± 2.74^dC^	58 ± 2.00^dA^	49 ± 4.85^dB^	29 ± 2.92^dC^
	500	89 ± 4.00^bA^	82 ± 4.64^cB^	55 ± 2.74^cC^	80 ± 2.74^cA^	71 ± 3.32^cB^	50 ± 3.54^cC^
	1000	100 ± 0.00^aA^	98 ± 2.00^bB^	70 ± 2.24^bC^	98 ± 2.00^bA^	96 ± 4.00^bB^	64 ± 3.32^bC^
	1500	100 ± 0.00^aA^	100 ± 0.00^aA^	80 ± 3.54^aB^	100 ± 0.00^aA^	100 ± 0.00^aA^	76 ± 2.92^aB^

a, b & c …. etc: There is no significant difference (p > 0.05) between any two means, within the same column have the same superscript letter; A, B & C … etc: There is no significant difference (p > 0.05) between any two means for the same solvent, within the same row have the same superscript letter. Five replicates were used for each concentration (20 larvae/replicate were used).

**Fig. 4:**
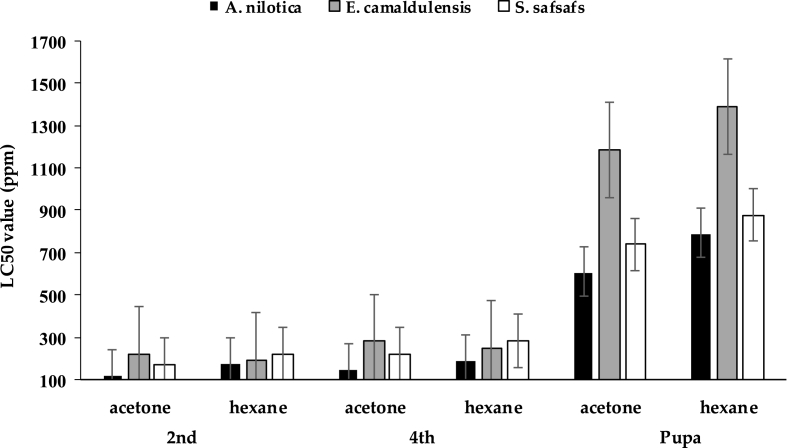
The mean number of larval mortalities induced by the effects of *Acacia nilotica, Eucalyptus camaldulensis, and Salix safsafs against* 3rd larval instars *Aedes aegypti*, 48 h post-exposure.

Similar to the response of *Cx. pipiens*, the results of this work showed that plant extracts, *A. nilotica, E. camaldulensis,* and *S. safsafs* effectively controlled the *Ae. aegypti* because 100% mortality was reached 24 and 48 h (2 days) post-treatments with acetone extracts of *A. nilotica* (LC_50_ = 181.79 and 123.57 ppm)*, E. camaldulensis,* (LC_50_ = 351.04 and 221.79 ppm), and *S. safsafs* (LC_50_ = 251.35 and 169.3 ppm) for 2nd larval instar at 1500 ppm (Table 4, Table 6). Larvicidal activity of *A. nilotica, E. camaldulensis,* and *S. safsafs* were effectively on the 4th larval instar, where the mortality reached 100, 90, and 100% at 24 h and 100% mortality for all plant extracts at 48 h post-treatment with LC_50_ values (213.62 and 152.78), (445.47 and 280.39), and (318.35 and 223.23 ppm), respectively (Table 4, Table 5). While at pupal stage, the mortality reached 70% (LC_50_ = 699.16), 52% (LC_50_ = 1250.02), and 65% (LC_50_ = 836.56) at 24 h and 73% (LC_50_ = 610.43), 54% (LC_50_ = 1184.54), and 68% (LC_50_ = 739.05) at 48 h post-treatment for *A. nilotica, E. camaldulensis,* and *S. safsafs*, 1500 ppm, respectively (Table 4, Table 5, Table 6, Fig. 4).

**Table 4:** Efficacy of *Acacia nilotica, Eucalyptus camaldulensis, and Salix safsafs* on *Aedes aegypti*, 24 h post-treatment.

Plant extract	Conc. (ppm)	Acetone			Hexane		
		2nd	4th	Pupae	2nd	4th	Pupae
*Acacia nilotica*	0	00.0±0^fA^	00.0±0^gA^	00.0±0^gA^	00.0±0^gA^	00.0±0^gA^	00.0±0^gA^
	62.5	14 ± 1.87^eA^	11 ± 1.87^fB^	5 ± 2.24^fC^	11 ± 1.87^fA^	6 ± 1.87^fB^	4 ± 1.87^fC^
	125	30 ± 1.58^dA^	24 ± 3.67^eB^	11 ± 3.32^eC^	24 ± 1.87^eA^	19 ± 1.87^eB^	9 ± 1.87^eC^
	250	64 ± 4.30^cA^	58 ± 3.39^dB^	26 ± 3.32^dC^	52 ± 2.55^dA^	45 ± 4.18^dB^	22 ± 5.15^dC^
	500	84 ± 1.87^bA^	78 ± 5.15^cB^	42 ± 4.36^cC^	81 ± 5.79^cA^	70 ± 5.24^cB^	38 ± 6.63^cC^
	1000	100 ± 0.00^aA^	98 ± 2.00^bB^	58 ± 7.84^bC^	98 ± 2.00^bA^	89 ± 4.30^bB^	54 ± 9.27^bC^
	1500	100 ± 0.00^aA^	100 ± 0.00^aA^	70 ± 4.18^aB^	100 ± 0.00^aA^	100 ± 0.00^aA^	66 ± 4.85^aB^
*Eucalyptus camaldulensis*	0	00.0±0^gA^	00.0±0^gA^	00.0±0^fA^	00.0±0^gA^	00.0±0^gA^	00.0±0^fA^
	62.5	7 ± 2.55^fA^	5 ± 1.58^fB^	00.0±0^fC^	10 ± 1.58^fA^	5 ± 1.58^fB^	00.0±0^fC^
	125	15 ± 3.16^eA^	13 ± 2.55^eB^	5 ± 1.58^eC^	22 ± 4.06^eA^	14 ± 2.92^eB^	4 ± 1.87^eC^
	250	36 ± 1.87^dA^	30 ± 1.58^dB^	18 ± 3.39^dC^	50 ± 2.24^dA^	34 ± 1.00^dB^	15 ± 4.74^dC^
	500	65 ± 2.24^cA^	52 ± 3.39^cB^	29 ± 2.92^cC^	74 ± 1.87^cA^	58 ± 4.36^cB^	24 ± 6.20^cC^
	1000	86 ± 3.67^bA^	73 ± 4.64^bB^	43 ± 3.74^bC^	95 ± 3.16^bA^	80 ± 5.70^bB^	38 ± 5.61^bC^
	1500	100 ± 0.00^aA^	90 ± 4.47^aB^	52 ± 3.00^aC^	100 ± 0.00^aA^	100 ± 0.00^aA^	47 ± 3.39^aB^
*Salix safsafs*	0	00.0±0^gA^	00.0±0^gA^	00.0±0^gA^	00.0±0^gA^	00.0±0^gA^	00.0±0^fA^
	62.5	10 ± 1.58^fA^	7 ± 1.22^fB^	2 ± 2.00^fC^	9 ± 1.00^fA^	4 ± 1.87^fB^	00.0±0^fC^
	125	21 ± 1.00^eA^	18 ± 2.55^eB^	9 ± 2.92^eC^	19 ± 2.45^eA^	12 ± 2.55^eB^	6 ± 2.92^eC^
	250	51 ± 5.10^dA^	41 ± 3.32^dB^	24 ± 2.45^dC^	48 ± 2.55^dA^	28 ± 1.22^dB^	19 ± 2.92^dC^
	500	75 ± 6.71^cA^	63 ± 4.36^cB^	37 ± 2.55^cC^	71 ± 5.57^cA^	50 ± 1.58^cB^	34 ± 4.00^cC^
	1000	95 ± 3.16^bA^	88 ± 4.06^bB^	53 ± 5.61^bC^	90 ± 3.54^bA^	75 ± 1.58^bB^	50 ± 6.52^bC^
	1500	100 ± 0.00^aA^	100 ± 0.00^aA^	65 ± 4.18^aB^	100 ± 0.00^aA^	95 ± 3.87^aB^	62 ± 5.39^aC^

a, b & c …. etc: There is no significant difference (p > 0.05) between any two means, within the same column have the same superscript letter; A, B & C … etc: There is no significant difference (p > 0.05) between any two means for the same solvent, within the same row have the same superscript letter. Five replicates were used for each concentration (20 larvae/replicate were used).

**Table 5:** Lethal concentrations of *Acacia nilotica, Eucalyptus camaldulensis,* and *Salix safsafs* against *Aedes aegypti*, 24 h post-treatment.

Stage	Plant extract	Solvents	LC_50_ (95% CL) *	LC_90_ (95% CL)	LC_95_ (95% CL)	Equation**	X^2^
2nd	*Acacia nilotica*	Acetone	181.7 (160.9–204.0)	547.1 (467.0–664.5)	747.7 (620.3–945.6)	2.678 ± 0.186	5.93
		Hexane	217.5 (193.2–243.8)	655.1 (559.6–793.8)	895.5 (744.0–1127.8)	2.676 ± 0.178	5.36
	*Eucalyptus camaldulensis*	Acetone	351.0 (305.8–403.3)	1433.7 (1145.7–1911.1)	2136.4 (1634.9–3026.9)	2.097 ± 0.157	2.52
		Hexane	249.7 (219.5–283.1)	874.7 (727.4–1100.3)	1247.9 (1002.6–1647.2	2.354 ± 0.165	6.35
	*Salix safsafs*	Acetone	251.3 (220.6–285.4)	892.8 (738.7–1131.7)	1278.9 (1021.1–1704.2)	2.328 ± 0.166	8.25
		Hexane	265.7 (234.8–299.6)	901.5 (759.6–1111.2)	1274.5 (1041.0–1639.7)	2.415 ± 0.159	6.00
4th	*Acacia nilotica*	Acetone	213.6 (189.4–239.7)	656.2 (559.5–797.2)	902.1 (747.6–1140.0)	2.629 ± 0.176	5.97
		Hexane	282.3 (250.4–317.4)	919.3 (778.0–1126.7)	1284.8 (1055.4–1640.2)	2.499 ± 0.163	4.98
	*Eucalyptus camaldulensis*	Acetone	445.4 (389.7–510.7)	1886.9 (1507.7–2507.4)	2841.09(2175.2–4003.8)	2.044 ± 0.144	2.41
		Hexane	363.5 (267.3–491.9)	1246.6 (961.2–2103.2)	1767.8 (1349.6–3250.2)	2.394 ± 0.158	11.70
	*Salix safsafs*	Acetone	318.3 (278.9–362.8)	1216.6 (994.0–1568.1)	1779.0 (1399.0–2418.8)	2.201 ± 0.156	1.29
		Hexane	438.1 (386.8–497.0)	1610.7 (1321.9–2061.4)	2329.6 (1843.7–3133.7)	2.266 ± 0.154	6.87
Pupae	*Acacia nilotica*	Acetone	699.1 (587.4–853.5)	4636.1 (3199.1–7703.6)	7926.0 (5092.2–14597.7)	1.559 ± 0.134	0.43
		Hexane	821.3 (685.3–1017.9)	5407.4 (3654.3–9303.6)	9225.8 (5793.7–17659.0)	1.565 ± 0.138	0.32
	*Eucalyptus camaldulensis*	Acetone	1250.0 (1010.8–1645.1)	8127.5 (5117.7–15851.2)	13817.7 (8026.1–30423.6)	1.576 ± 0.154	4.39
		Hexane	1539.6 (1210.8–2133.0)	10441.3 (6202.9–22658.3)	17964.5 (9773.3–44649.3)	1.541 ± 0.161	3.60
	*Salix safsafs*	Acetone	836.5 (699.6–1034.0)	5291.8 (3604.7–8995.1)	8926.8 (5661.6–16829.8)	1.599 ± 0.140	1.93
		Hexane	943.0 (794.5–1157.7)	5050.2 (3526.5–8288.8)	8126.6 (5319.3–14648.5)	1.758 ± 0.154	3.28

* LC50 values = lethal concentration that kills 50% of the exposed larvae; (95%CL) = lower and upper confidence limit; ** Regression line equation; X2 = chi-square; Significant at P < 0.05 level.

**Table 6:** Efficacy of *Acacia nilotica, Eucalyptus camaldulensis, and Salix safsafs* on *Aedes aegypti*, 48 h post-treatment.

Plant extract	Conc. (ppm)	Acetone			Hexane		
		2nd	4th	Pupae	2nd	4th	Pupae
*Acacia nilotica*	0	00.0±0^fA^	00.0±0^fA^	00.0±0^gA^	00.0±0^fA^	00.0±0^fA^	00.0±0^fA^
	62.5	22 ± 3.39^eA^	16 ± 2.92^eB^	8 ± 2.00^fC^	14 ± 1.87^eA^	10 ± 4.18^eB^	4 ± 1.87^eC^
	125	45 ± 4.74^dA^	39 ± 5.34^dB^	14 ± 4.30^eC^	28 ± 2.55^dA^	31 ± 4.30^dB^	9 ± 2.45^dC^
	250	82 ± 3.39^cA^	74 ± 5.34^cB^	29 ± 4.30^dC^	64 ± 2.92^cA^	60 ± 7.58^cB^	22 ± 5.39^C^
	500	98 ± 1.22^bA^	88 ± 4.06^bB^	45 ± 5.24^cC^	88 ± 2.55^bA^	84 ± 4.58^bB^	38 ± 6.63^cC^
	1000	100 ± 0.00^aA^	100 ± 0.00^aA^	61 ± 8.57^bB^	100 ± 0.00^aA^	100 ± 0.00^aA^	55 ± 9.49^bB^
	1500	100 ± 0.00^aA^	100 ± 0.00^aA^	73 ± 5.15^aB^	100 ± 0.00^aA^	100 ± 0.00^aA^	68 ± 5.61^aB^
*Eucalyptus camaldulensis*	0	00.0±0^gA^	00.0±0^gA^	00.0±0^gA^	00.0±0^fA^	00.0±0^gA^	00.0±0^gA^
	62.5	13 ± 3.00^fA^	9 ± 2.92^fB^	2 ± 2.00^fC^	13 ± 1.22^eA^	9 ± 1.87^fB^	00.0±0^fC^
	125	29 ± 2.92^eA^	21 ± 2.92^eB^	7 ± 2.55^eC^	30 ± 5.48^dA^	24 ± 2.45^eB^	4 ± 1.87^eC^
	250	52 ± 3.39^dA^	43 ± 3.39^dB^	20 ± 2.74^dC^	59 ± 4.85^cA^	50 ± 3.16^dB^	16 ± 4.00^dC^
	500	75 ± 6.12^cA^	68 ± 8.75^cB^	31 ± 2.92^cC^	80 ± 2.74^bA^	70 ± 4.18^cB^	25 ± 5.24^cC^
	1000	94 ± 3.67^bA^	89 ± 4.85^bB^	45 ± 3.87^bC^	100 ± 0.00^aA^	94 ± 3.67^bB^	39 ± 5.10^bC^
	1500	100 ± 0.00^aA^	100 ± 0.00^aA^	54 ± 4.00^aB^	100 ± 0.00^aA^	100 ± 0.00^aA^	51 ± 3.67^aB^
*Salix safsafs*	0	00.0±0^gA^	00.0±0^gA^	00.0±0^gA^	00.0±0^gA^	00.0±0^gA^	00.0±0^gA^
	62.5	18 ± 4.90^eA^	13 ± 2.55^fB^	5 ± 2.24^fC^	11 ± 1.00^fA^	8 ± 2.00^fB^	00.0±0^fC^
	125	36 ± 7.65^dA^	28 ± 5.15^eB^	12 ± 4.64^eC^	24 ± 2.92^eA^	20 ± 3.54^eB^	7 ± 2.55^eC^
	250	64 ± 9.67^cA^	56 ± 3.67^dB^	27 ± 4.06^dC^	55 ± 4.18^dA^	40 ± 3.54^dB^	22 ± 3.39^dC^
	500	82 ± 5.15^bA^	76 ± 2.92^cB^	40 ± 4.18^cC^	78 ± 3.39^cA^	69 ± 5.57^cB^	35 ± 3.54^cC^
	1000	100 ± 0.00^aA^	95 ± 3.16^bB^	56 ± 7.31^bC^	96 ± 2.45^bA^	90 ± 2.74^bB^	52 ± 6.44^bC^
	1500	100 ± 0.00^aA^	100 ± 0.00^aA^	68 ± 5.15^aB^	100 ± 0.00^aA^	100 ± 0.00^aA^	64 ± 5.10^aB^

a, b & c …. etc: There is no significant difference (P > 0.05) between any two means, within the same column have the same superscript letter; A, B & C … etc: There is no significant difference (P > 0.05) between any two means for the same solvent, within the same row have the same superscript letter. Five replicates were used for each concentration (20 larvae/replicate were used).

The hexane extracts of *A. nilotica, E. camaldulensis,* and *S. safsafs* were well controlled against *Ae. aegypti* for up to 48 h, where the mortality reached 100% for 2nd and 4th larval instar with LC_50_ values (179.73, 194.35, and 221.81 ppm), and (192.26, 247.58, and 283.59) at 1500 ppm, respectively. While plant extracts, *A. nilotica, E. camaldulensis,* and *S. safsafs* were less effective on pupae of *Ae. aegypti*, where the mortality reached 68 (LC_50_ = 790.75), 51 (LC_50_ = 1312.14), and 64% (LC_50_ = 878.40). This work indicated that complete mortalities were reached 24 and 48 h PT of the *Cx. pipiens* and *Ae. aegypti* with 1500 ppm extracts of *Salix safsafs* (LC_50_ values PT with acetone extract were 261.84, 318.35 and 175.99, 223.23 for *Cx. pipiens* and *Ae. aegypti*, respectively, whereas those of hexane extracts were 357.30, 438.13 and 257.39, 283.59 ppm, respectively.

The results showed that all plant extracts in this study showed moderate to high toxicity effects against mosquito species *Cx. pipiens* and *Ae. aegypti*, and acetone extracts were more effective than hexane extracts.

### 3.2 Effect of sub-lethal on larval development

The sub-lethal concentration effects of *A. nilotica, E. camaldulensis,* and *S. safsafs* extracts on mosquito larval development elongation persisted for 14, 10.5 and 14.8 days for acetone extracts (LC_50_ = 175.71, 374.15 and 261.84 ppm) and 10.4, 9.8, and 12.6 days for hexane extracts (LC_50_ = 249.31, 341.29, and 357.30 ppm) with cumulative larvicidal mortality rates of *Cx. pipiens* 87.8, 75.6, and 81.1 for acetone extracts, and 75.6, 77.8 and 87.8% for hexane extracts, respectively (Table 7). The results also showed that those effects were obvious on *Ae. aegypti* larvae, where it took 16, 14.7, and 18.5 days for the larvae to reach the adults in acetone extracts, and 13.6, 14.5, and 16.8 days in hexane extracts, respectively. The cumulative larvicidal mortality rates were 92.2, 81.1, 80, and 80, 82.2 and 75.6%, respectively (Table 7). For *E. camaldulensis* extract, it had the least effect on the elongation of mosquito larval growth in *Cx. pipiens* or *Ae. aegypti* larvae, where the experiment took 10.5 and 14.7 days to reach adult with a mortality rate of 75.6 and 81.1%, respectively. During the experiments, it was observed that the larvae were able to develop and reach the adult stage. The plant extracts that significantly reduced the adult emergence rate of *Cx. pipiens* and *Ae. aegypti* was *A. nilotica* plant, either in acetone (5.6 and 8.9 days) or hexane (12.2 and 15.6 days) extracts (Table 7).

**Table 7:** Effects of sub-lethal concentrations (LC_50_) of *Acacia nilotica, Eucalyptus camaldulensis,* and *Salix safsafs* on development mosquito larvae.

Species	Plant extract	Duration (days)		Death larvae (%)		Death pupae (%)		Emergence adults (%)	
		Acetone	Hexane	Acetone	Hexane	Acetone	Hexane	Acetone	Hexane
*Culex pipiens*	Control	5.6 ± 0.15^dA^	5.6 ± 0.15^cA^	7.8 ± 1.11^cA^	7.8 ± 1.11^bA^	1.1 ± 1.11^dA^	1.1 ± 1.11^cA^	94.4 ± 1.11^aA^	94.4 ± 1.11^aA^
	*A. nilotica*	14.0 ± 0.01^bA^	10.4 ± 0.23^bB^	87.8 ± 2.94^aA^	75.6 ± 7.29^aB^	18.9 ± 2.94^aA^	15.6 ± 1.11^aB^	5.6 ± 1.11^cB^	12.2 ± 2.94^cA^
	*E. camaldulensis*	10.5 ± 0.33^cA^	9.8 ± 0.81^bA^	75.6 ± 2.94^bA^	77.8 ± 2.94^aA^	10.0 ± 1.92^cA^	11.1 ± 1.11^bA^	12.2 ± 2.94^bA^	13.3 ± 1.93^cA^
	*S. safsafs*	14.8 ± 0.56^aA^	12.6 ± 0.69^aB^	81.1 ± 2.94^abA^	75.6 ± 1.11^aB^	14.4 ± 1.11^bA^	12.2 ± 2.94^abA^	14.4 ± 2.94^bB^	20.0 ± 0.00^bA^
*Aedes aegypti*	Control	7.0 ± 0.40^cA^	7.0 ± 0.40^cA^	11.1 ± 2.94^cA^	11.1 ± 2.94^bA^	4.5 ± 2.22^cA^	4.5 ± 2.22^bA^	91.1 ± 1.11^aA^	91.1 ± 1.11^aA^
	*A. nilotica*	16.0 ± 0.71^bA^	13.6 ± 0.15^bB^	92.2 ± 4.84^aA^	80.0 ± 5.09^aB^	25.6 ± 2.94^aA^	17.8 ± 2.94^aB^	8.9 ± 2.22^cB^	15.6 ± 2.94^cA^
	*E. camaldulensis*	14.7 ± 1.11^bA^	14.5 ± 1.20^bA^	81.1 ± 5.88^bA^	82.2 ± 6.76^aA^	15.6 ± 1.11^bA^	13.3 ± 3.33^aA^	18.9 ± 4.84^bA^	21.1 ± 2.94^bcA^
	*S. safsafs*	18.5 ± 1.07^aA^	16.8 ± 0.72^aB^	80.0 ± 3.85^bA^	75.6 ± 2.22^aA^	20.0 ± 3.85^abA^	15.6 ± 2.22^aB^	20.0 ± 0.00^bB^	24.4 ± 4.44^bA^

a, b & c …. etc: There is no significant difference (p > 0.05) between any two means, within the same column have the same superscript letter; A, B & C … etc: There is no significant difference (p > 0.05) between any two means for the same solvent, within the same row have the same superscript letter. Three replicates were used for each concentration (30 larvae/replicate were used).

### 3.3 Oviposition bioassay

To evaluate the effectiveness of the effect of *A. nilotica, E. camaldulensis,* and *S. safsafs* plant extracts on the reduction of egg laying and hatching percentages in *Cx. pipiens* and *Ae. aegypti* females at LC_50_ and LC_90_ concentrations, the results showed a clear effect of the plant extracts in reducing the rate of egg laying by female mosquitoes at the selected concentrations. The average number of eggs laid by female *Cx. pipiens* in cups was 28.5, 72, and 80.7 eggs at the LC_50_ value and 8.1, 34.7, and 45.5 eggs at the LC_90_ value. Similarly, *Ae. aegypti* female mosquitoes laid fewer eggs when exposed to LC_50_ or LC_90_ concentrations. The results also showed that the rate of hatching was affected, especially at the high concentration (LC_90_) treated with it, as the rate of hatching was 4.4, 17.8, and 22 for *Cx. pipiens* females, and 9, 18.7, and 22.9 for female *Ae. aegypti* treated with A*. nilotica, E. camaldulensis, and S. safsafs* plant extracts, respectively (Table 8). The paired *t*-test revealed that these outcomes were statistically significant (P > 0.05).

**Table 8:** Efficacy of plant extracts on oviposition rates of female *Culex pipiens* and *Aedes aegypti* after exposure to LC_50_ and LC_90_ values.

Mosquito species	Plant oil	Conc. (ppm)	Mean number of eggs ± SD		P value	Number of eggs laid/female	Hatching %
			Treated	Untreated			
*Culex pipiens*	*Acacia nilotica*	500	524.0 ± 60.1*	2780.0 ± 122.1	0.000	28.5	15.4
		1000	139.0 ± 46.4*	2590.0 ± 62.8	0.000	**8.1**	**4.4**
	*Eucalyptus camaldulensis*	500	1080.0 ± 46.4*	2922.0 ± 76.3	0.021	72.0	37.0
		1000	520.0 ± 31.8*	2864.0 ± 44.1	0.000	**34.7**	**17.8**
	*Salix safsafs*	500	1210.0 ± 152.2*	3100.0 ± 90.5	0.001	80.7	39.0
		1000	682.0 ± 76.3*	3010.0 ± 150.3	0.008	**45.5**	**22.0**
*Aedes aegypti*	*Acacia nilotica*	500	133.0 ± 14.4*	564.0 ± 24.2	0.000	8.9	23.6
		1000	51.0 ± 7.2*	508.0 ± 43.3	0.009	**3.4**	**9.0**
	*Eucalyptus camaldulensis*	500	213.0 ± 15.2*	520.0 ± 31.8	0.002	14.2	41.0
		1000	97.0 ± 19.6*	530.0 ± 55.6	0.021	**6.5**	**18.7**
	*Salix safsafs*	500	259.0 ± 12.8*	586.0 ± 40.9	0.011	17.3	44.2
		1000	134.0 ± 14.6*	560.0 ± 58.1	0.015	**8.9**	**22.9**

* The significant differences between the treated and untreated group were determined by paired *t*-test (P < 0.05).

### 3.4 Larvicidal activity of plant extracts as photosensitizer

All plant extracts in this study showed normal effects against *Cx. pipiens* and *Ae. aegypti* larvae after 24 h post-treatment, as shown in the larvicidal activity Tables (1–2, 5–6) at the same concentration. *A. nilotica* plant extracts showed high toxic effects on mosquito larvae, where the mortality % reached 36% and 93% at 62.5 and 125 ppm for *Cx. pipiens* in acetone, and 28% and 80% ppm in hexane extracts after sunlight exposure. In shadow light, the mortality % reached 13%, 34%, 8%, and 21% for *A. nilotica* acetone and hexane at 62.5 and 125 ppm, respectively (Table 9). Similarly, *Ae. aegypti* larvae mortality rates were 27% and 79% in sunlight for acetone and 22% and 70% for hexane, respectively, while in shadow light the mortality rates were 11 and 25% for acetone and 6 and 18% for hexane extracts (Table 9). Data showed that, at 125 ppm, the effects of *E. camaldulensis* and *S. safsafs* plant extracts were not affected on larval mortality either in sunlight or under shadow lights.

**Table 9:** Larvicidal activity of plant extracts as photosensitizer against the 4th *Culex pipiens* and *Aedes aegypti*, 24 h post-treatment.

Species	Plant extract	Sunlight				Shadow light			
		Acetone		Hexane		Acetone		Hexane	
*Culex pipiens*	Conc. (ppm)	62.5	125	62.5	125	62.5	125	62.5	62.5
	Control	2.0 ± 1.22^aC^	2.0 ± 1.22^aC^	2.0 ± 1.22^aC^	2.0 ± 1.22^aC^	1.0 ± 1.00^aC^	1.0 ± 1.00^aC^	0.0 ± 0.0^aC^	2.0 ± 1.22^aC^
	*A. nilotica*	36.0 ± 2.92^aB^	93.0 ± 5.39^aA^	28.0 ± 4.64^aB^	80.0 ± 5.92^aA^	13.0 ± 2.55^aB^	34.0 ± 3.32^aA^	8.0 ± 2.55^aB^	36.0 ± 2.92^aB^
	*E. camaldulensis*	11.0 ± 1.87^cB^	21.0 ± 3.32^cA^	13.0 ± 1.22^bB^	26.0 ± 1.87^bA^	8.0 ± 2.55^bB^	16.0 ± 3.67^cA^	8.0 ± 2.00^aB^	11.0 ± 1.87^cB^
	*S. safsafs*	14.0 ± 2.45^bB^	25.0 ± 3.16^bA^	11.0 ± 1.87^cB^	23.0 ± 2.00^cA^	8.0 ± 1.22^bB^	21.0 ± 2.45^bA^	5.0 ± 1.58^bB^	14.0 ± 2.45^bB^
*Aedes aegypti*	Control	1.0 ± 1.00^aC^	1.0 ± 1.00^aC^	1.0 ± 1.00^aC^	1.0 ± 1.00^aC^	0.0 ± 0.0^aC^	0.0 ± 0.0^aC^	0.0 ± 0.0^aC^	1.0 ± 1.00^aC^
	*A. nilotica*	27.0 ± 2.55^aB^	79.0 ± 8.43^aA^	22.0 ± 4.64^aB^	70.0 ± 5.48^aA^	11.0 ± 1.87^aB^	25.0 ± 3.54^aA^	6.0 ± 1.87^aB^	27.0 ± 2.55^aB^
	*E. camaldulensis*	9.0 ± 1.00^cB^	20.0 ± 1.58^cA^	11.0 ± 1.87^bB^	25.0 ± 1.58^bA^	5.0 ± 1.58^cB^	14.0 ± 4.00^cA^	4.0 ± 1.87^bB^	9.0 ± 1.00^cB^
	*S. safsafs*	11.0 ± 1.87^bB^	25.0 ± 4.74^bA^	9.0 ± 1.00^cB^	22.0 ± 2.55^cA^	7.0 ± 2.00^bB^	19.0 ± 1.87^bA^	4.0 ± 1.87^bB^	11.0 ± 1.87^bB^

a, b & c …. etc: There is no significant difference (p > 0.05) between any two means, within the same column have the same superscript letter; A, B & C … etc: There is no significant difference (p > 0.05) between any two means for the same solvent, within the same row have the same superscript letter. Five replicates were used for each concentration (20 larvae/replicate were used).

### 3.5 Phytochemical analysis

**Fig. 5:**
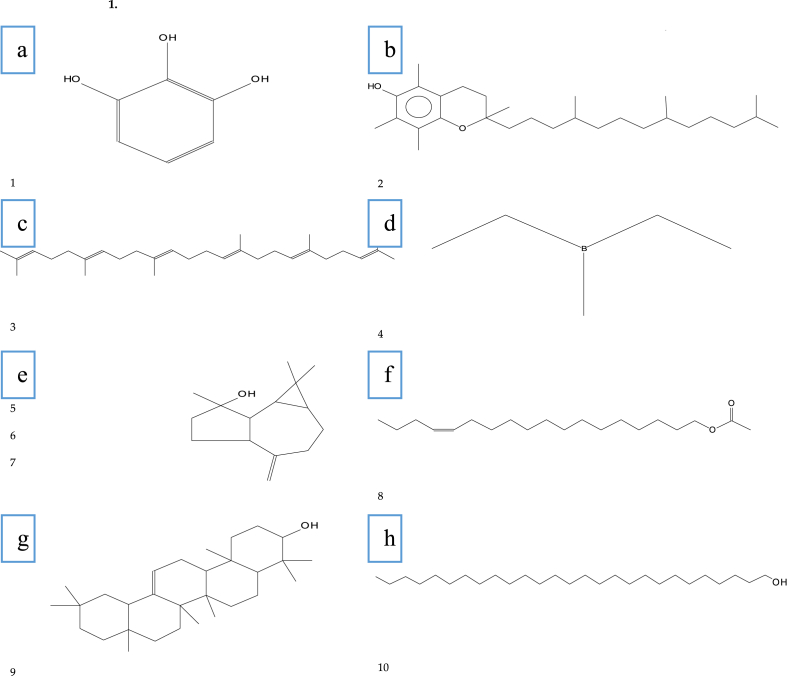
Structure of the main compounds in plant extracts: *A. nilotica* (a: 1,2,3-BENZENETRIOL, b: 2,3-DI-N-NONYLANTHRAENE, c: Squalene, d:17-Pentatriacontene), *E. camaldulensis* (e:1H-Cycloprop[e]azulen-7-ol, decahydro-1,1,7-trimethyl-4-methylene-, [1ar-(1aà,4aà,7á,7aá,7bà)]-, f: Octacosanal) and *S. safsafs* (g: α-Amyrin, and h: 1-Heptacosanol).

Phytochemical analysis was done with GC–MS chromatogram for acetone and hexane extracts of *A. nilotica, E. camaldulensis,* and *S. safsafs*. These phytochemical compounds recognized by relating their peak retention time, peak area (%), and mass spectral fragmentation patterns to that of the known compounds described by the National Institute of Standards and Technology (NIST) library (Table 10, Table 11, Table 12, Table 13, Table 14, Table 15). Results showed that phytochemical analysis for *A. nilotica* acetone and hexane extracts indicating 25 and 18 phytochemical compounds, where the main chemical compounds are 2,3-DI-N-Nonylanthraene (13.69%), 1,2,3-Benzenetriol (13.46%), 9,12,15-Octadecatrienoic acid, (Z,Z,Z)- (11.16%), Squalene (8.79%), n-Hexadecanoic acid (6.86%), and 9-Octadecenoic acid, methyl ester, (E)- (6.40%) for *A. nilotica* acetone extract and dl-à-Tocopherol (16.88%), Squalene (14.74), 17-Pentatriacontene (10.58%), Stigmasterol (9.75%), and 9,12,15-Octadecatrienoic acid, (Z,Z,Z)- (7.46) for *A. nilotica* hexane (Table 10, Table 11), (Fig. 5).

**Table 10:** The major chemical constituents of *Acacia nilotica* acetone extracts.

No.	RT	Compounds (100%)	M. F.	M. W.	Area (%)	R.I*	Classification
1	9.73	Catechol	C_6_H_6_O_2_	110	5.39	1205	fatty acid
2	14.19	1,2,3-Benzenetriol	C_6_H_6_O_3_	126	13.46	1386	alkaline pyrogallol
3	20.39	Mome Inositol	C_7_H_14_O_6_	194	1.21	NA	heterocyclic compound
4	22.88	Tetradecanoic Acid	C_14_H_28_O_2_	228	0.41	1769	carboxylic acid
5	24.42	2,6,10-Trimethyl,14-Ethylene-14-Pentadecne	C_20_H_38_	278	3.02	1774	Palmitic Acid
6	25.28	3,7,11,15-Tetramethyl-2-hexadecen-1-ol	C_20_H_40_O	296	1.88	2045	USFA
7	26.19	Hexadecanoic acid, Methyl ester	C_17_H_34_O_2_	270	2.42	1926	FAME
8	26.99	n-Hexadecanoic acid	C_16_H_32_O_2_	256	6.86	1968	Palmitic Acid
9	27.69	Ethylene brassylate	C_15_H_26_O_4_	270	1.17	1989	macrocyclic musk
10	29.33	9,12-Octadecadienoic acid (Z,Z)-, methyl ester	C_19_H_34_O_2_	294	4.53	2092	fatty acid
11	29.47	9-Octadecenoic acid, methyl ester, (E)-	C_19_H_36_O_2_	296	6.40	2110	MUSFA
12	29.98	Octadecanoic acid, Methyl Ester	C_19_H_38_O_2_	298	0.64	2077	FAME
13	30.26	9,12,15-Octadecatrienoic acid, (Z,Z,Z)-	C_18_H_30_O_2_	278	11.16	2139	fatty acid
14	30.68	Octadecanoic acid	C_18_H_36_O_2_	284	3.00	2172	sesquiterpene
15	31.95	Fluoromethacin	C_19_H_16_FNO_4_	341	2.55	NA	methyl fluoride
17	37.33	Benz[E]acephenanthrylen-12-OL	C_20_H_12_O	268	1.53	NA	Fluorescein
18	37.77	17-Pentatriacontene	C_35_H_70_	490	1.36	3508	wax
19	38.82	2-Hydroxy-3-[(9E)-9-Octadecenoyloxy]Propyl (9E)-9-Octadecenoate	C_39_H_72_O_5_	620	1.61	4411	MUSFA
20	39.81	Squalene	C_30_H_50_	410	8.79	2832	triterpene
21	40.61	Dotriacontane	C_32_H_66_	450	3.66	3202	alkanes
22	42.06	6,7-Epoxypregn-4-ene-9,11,18-triol-3,20-dione, 11,18-diacetate	C_25_H_32_O_8_	460	1.97	3103	triterpene
23	42.81	17-Pentatriacontene	C_35_H_70_	490	2.13	3508	wax
24	43.03	2,3-DI-N-NONYLANTHRAENE	C_32_H_46_	430	**13.69**	3138	polyethylene glycol
25	44.03	1-Heptatriacotanol	C_37_H_76_O	536	1.12	3942	sesquiterpene

*R.I: ; Unsaturated fatty acids (USFA); fatty acid methyl ester (FAME); monounsaturated fatty acid (MUSFA).

**Table 11:** The major chemical constituents of *Acacia nilotica* hexane extracts.

No.	RT	Compounds (100%)	M. F.	M. W.	Area (%)	R.I	Classification
1	13.09	1,2,3-Propanetriol, Triacetate	C_9_H_14_O_6_	218	1.17	1344	triglyceride
2	21.56	Docosane	C_22_H_46_	310	1.49	2208	alkane
3	26.20	Hexadecanoic acid, Methyl Ester	C_17_H_34_O_2_	270	1.90	1926	FAME
4	26.94	Hexadecanoic acid	C_16_H_32_O_2_	256	4.56	1968	Palmitic Acid
5	29.32	7,10-Octadecadienoic acid, methyl ester	C_19_H_34_O_2_	294	1.40	2097	methyl esters
6	29.46	9-Octadecenoic acid (Z)-, methyl ester	C_19_H_36_O_2_	296	2.72	2105	FAME
7	30.18	9,12,15-Octadecatrienoic acid, (Z,Z,Z)-	C_18_H_30_O_2_	278	7.46	2139	fatty acid
8	30.29	9-Octadecenoic Acid (Z)-	C_18_H_34_O_2_	282	1.36	2141	MUSFA
9	30.65	Octadecanoic acid	C_18_H_36_O_2_	284	1.86	2172	sesquiterpene
10	36.84	Stigmasterol	C_29_H_48_O	412	**9.75**	3343	phytosterols
11	39.81	Squalene	C_30_H_50_	410	**14.74**	2832	triterpene
12	39.90	Lupeol	C_30_H_50_O	426	4.92	3270	triterpene
13	40.04	9-Hexadecenoic acid, 9-octadecenyl ester, (Z,Z)-	C_34_H_64_O_2_	504	0.78	3503	fatty aldehyde
14	40.61	Dotriacontane	C_32_H_66_	450	**9.69**	3202	alkanes
15	42.05	Ethanol, 2-(9-Octadecenyloxy)-, (Z)-	C_20_H_40_O_2_	312	6.57	2336	USFA
16	42.81	17-Pentatriacontene	C_35_H_70_	490	**10.58**	3508	wax
17	43.02	dl-α-Tocopherol	C_29_H_50_O_2_	430	**16.88**	3138	triterpene
18	43.32	Ethyl iso-allocholate	C_26_H_44_O_5_	436	2.17	3094	triterpene

**Table 12:** The major chemical constituents of *Eucalyptus camaldulensis* acetone extracts.

No.	RT	Compounds (100%)	M. F.	M. W.	Area (%)	R.I	Classification
1	4.91	à-Phellandrene	C_10_H_16_	136	0.64	136	monoterpene
2	5.27	Benzene, 1-methyl-3-(1-methylethyl)-	C_10_H_14_	134	4.44	134	monoterpene
3	5.41	1,4-cyclohexadiene, 1-Methyl-4-(1-Methylethyl)-	C_10_H_16_	154	2.73	154	monoterpene
4	8.94	Terpinen-4-ol	C_10_H_18_O	154	2.34	154	cycloalkene
5	9.17	2-Cyclohexen-1-one, 4-(1-methylethyl)-	C_9_H_14_O	138	**6.31**	138	triglyceride
6	10.08	exo-2-Hydroxycineole	C_10_H_18_O_2_	170	0.32	170	cycloalkene
7	10.52	Benzaldehyde, 4-(1-methylethyl)-	C_10_H_12_O	148	1.04	148	aldehyde
8	10.68	Limonene Dioxide 1	C_10_H_16_O_2_	168	1.37	168	monoterpene
9	11.82	Benzenemethanol, 4-(1-Methylethyl)-	C_10_H_14_O	150	0.97	150	flavonoid
10	11.92	Cyclohexene, 1,5,5-trimethyl-6-acetylmethyl-	C_12_H_20_O	180	0.38	180	cycloalkene
11	12.55	Bicyclo(3.1.1)heptane-2,3-diol, 2,6,6-trimethyl-	C_10_H_18_O_2_	170	4.49	170	cycloalkene
12	12.93	7a-Methyl-3-methylenehexahydrobenzofuran-2-one	C_10_H_14_O_2_	166	1.07	166	monoterpene
13	13.71	1-Cyclohexene-1-methanol, à,à,4-trimethyl-	C_10_H_18_O	266	2.82	266	cycloalkene
14	14.26	2-Octen-1-ol, 3,7-dimethyl-, isobutyrate, (Z)-	C_14_H_26_O_2_	226	0.39	226	fatty alcohol
15	14.82	2-Pentyl-cyclohexane-1,4-diol	C_11_H_22_O_2_	186	0.42	186	phenol
16	16.77	6-Nonenal, 3,7-dimethyl-	C_11_H_20_O	168	**6.50**	168	Phenol
17	17.65	5,6,6-Trimethyl-5-(3-oxobut-1-enyl)-1-oxaspiro[2.5]octan-4-one	C_14_H_20_O_3_	236	0.45	236	phenol
18	18.12	2-Cyclohexen-1-one, 3-(hydroxymethyl)-6-(1-methylethyl)-	C_10_H_16_O_2_	168	0.73	168	monoterpene
19	18.73	1H-Cycloprop[e]azulen-7-ol, decahydro-1,1,7-trimethyl-4-methylene-, [1ar-(1aà,4aà,7á,7aá,7bà)]-	C_15_H_24_O	220	**13.64**	220	sesquiterpene
20	18.89	Alloaromadendrenoxid-(2)	C_15_H_24_O	220	0.54	220	sesquiterpene
21	19.35	Ledol	C_15_H_26_O	222	0.29	222	sesquiterpene
22	20.50	Caryophyllene oxide	C_15_H_24_O	220	2.05	220	sesquiterpene
23	21.92	1,1,4,7-Tetramethyldecahydro-1H-cyclopropa[e]azulene-4,7-diol	C_15_H_26_O_2_	238	4.82	238	sesquiterpene
24	22.14	Ylangenal	C_15_H_22_O	218	2.78	218	sesquiterpene
25	23.62	Isoaromadendrene epoxide	C_15_H_2_4O	220	0.61	220	sesquiterpene
26	24.43	1-Heptatriacotanol	C_37_H_76_O	536	2.85	536	sesquiterpene
27	24.51	2-Pentadecanone, 6,10,14-trimethyl-	C_18_H_36_O	268	0.31	268	sesquiterpene
28	24.80	2-(4a,8-Dimethyl-6-oxo-1,2,3,4,4a,5,6,8a-octahydro-naphthalen-2-yl)-propionaldehyde	C_15_H_22_O_2_	234	1.86	234	sesquiterpene
29	25.46	4,7-Octadecadiynoic acid, methyl ester	C_19_H_30_O_2_	290	0.58	290	fatty acid
30	25.56	Corymbolone	C_15_H_24_O_2_	236	0.65	236	sesquiterpene
31	26.20	Pentadecanoic Acid, 14-Methyl-, Methyl Ester	C_17_H_34_O_2_	270	0.36	270	FAME
32	26.96	Hexadecanoic acid	C_16_H_32_O_2_	256	3.96	256	palmitic Acid
33	28.31	[2-(5-Hydroxypent-2-ynyl)-3-oxocyclopentyl]thioacetic acid, S-t-butyl ester	C_16_H_24_O_3_S	296	0.58	296	carboxylic acid
34	29.47	10-Octadecenoic acid, methyl ester	C_19_H_36_O_2_	296	1.90	296	FAME
35	30.20	9-Octadecenoic Acid (Z)-	C_18_H_34_O_2_	282	1.68	282	MUSFA
36	36.26	Dotriacontane	C_32_H_66_	450	1.12	450	alkanes
37	36.63	1,2-Benzenedicarboxylic Acid	C_24_H_38_O_4_	390	0.29	390	FAMS
38	38.93	Heptacosane	C_27_H_56_	380	4.46	380	alkane
39	39.75	Isochiapin B	C_19_H_22_O_6_	346	0.36	346	sesquiterpene lactone
40	42.06	Ethanol, 2-(9-octadecenyloxy)-, (Z)-	C_20_H_40_O_2_	312	**8.86**	312	USFA
41	42.81	17-Pentatriacontene	C_35_H_70_	490	**7.51**	490	wax
42	43.03	Vitamin E	C_29_H_50_O_2_	430	0.53	430	aldehyde

* Fatty acid methyl ester (FAME); monounsaturated fatty acid (MUSFA); phthalic acid monoester (FAMS).

**Table 13:** The major chemical constituents of *Eucalyptus camaldulensis* hexane extracts.

No.	RT	Compounds (100%)	M. F.	M. W.	Area (%)	R.I	Classification
1	5.26	o-Cymene	C_10_H_14_	134	2.96	1022	monoterpene
2	18.72	(−)-Spathulenol	C_15_H_24_O	220	2.87	1577	sesquiterpene
3	26.95	Hexadecanoic Acid	C_16_H_32_O_2_	256	1.54	1968	palmitic Acid
4	29.33	8,11-Octadecadienoic acid, methyl ester	C_19_H_34_O_2_	294	0.61	2112	methyl esters
5	29.47	10-octadecenoic Acid, Methyl Ester	C_19_H_36_O_2_	296	0.63	2105	FAME
6	37.86	α-Sitosterol	C_29_H_50_O	414	5.46	3321	triterpene
7	38.79	Benzoic Acid, 2-(2,3-Dihydro-2,6-Dihydroxy-7-Methoxynaphtho [1,8-bc]pyran-9-YL)-5-et Henyl-3-Methoxy-, (R)-	C_23_H_20_O_7_	408	**12.11**	2958	ACA
8	38.93	Dotriacontane	C_32_H_66_	450	1.22	3202	alkanes
9	39.12	Betulinaldehyde	C_23_H_22_O_7_	440	7.02	3629	triterpene
10	39.42	Isochiapin B	C_19_H_22_O_6_	346	1.59	NA	STL
11	39.55	Betulin	C_30_H_50_O_2_	442	2.49	3761	triterpene
12	40.05	Ethanol, 2-(9-Octadecenyloxy)-,(Z)-	C_20_H_40_O_2_	312	**8.14**	2336	SFA
13	40.61	17-Pentatriacontene	C_35_H_70_	490	**9.73**	3508	wax
14	40.98	Ethyl iso-allocholate	C_26_H_44_O_5_	436	2.17	3094	triterpene
15	41.90	Olean-12-ene-3,28-diol, (3á)-	C_30_H_50_O_2_	442	1.87	3128	triterpene
16	42.06	Octacosanal	C_28_H_56_O	408	**28.72**	3036	fatty aldehyde
17	42.81	17-Pentatriacontene	C_35_H_70_	490	**10.42**	3508	wax
18	42.86	Ylangenal	C_15_H_22_O	218	0.44	2341	sesquiterpene

* Fatty acid methyl esters (FAME); aromatic carboxylic acid (ACA); sesquiterpene lactone (STL); saturated fatty acids (SFA).

**Table 14:** The major chemical constituents of *Salix safsafs* acetone extracts.

No.	RT	Compounds (100%)	M. F.	M. W.	Area (%)	R.I	Classification
1	13.10	1,2,3-Propanetriol, Triacetate	C_9_H_14_O_6_	218	0.48	1344	triglyceride
2	22.87	6-Hydroxy-4,4,7a-trimethyl-5,6,7,7a-tetrahydrobenzofuran-2(4H)-one	C_11_H_16_O_3_	196	0.44	1784	flavonoids
3	24.43	2,6,10-Trimethyl,14-Ethylene-14-Pentadecne	C_20_H_38_	278	**4.51**	1837	palmitic acid
4	24.51	2-Pentadecanone, 6,10,14-Trimethyl-	C_18_H_36_O	268	0.72	1844	sesquiterpene
5	24.93	3,7,11,15-Tetramethyl-2-hexadecen-1-ol	C_20_H_40_O	296	2.18	2116	USFA
6	26.20	Hexadecanoic Acid, Methyl Ester	C_17_H_34_O_2_	270	1.00	1878	FAME
7	26.99	n-Hexadecanoic acid	C_16_H_32_O_2_	256	**9.91**	1968	palmitic acid
8	29.33	9,12-Octadecadienoic acid (Z,Z)-, methyl ester	C_19_H_34_O_2_	294	2.98	2092	fatty acid
9	30.20	9,12,15-Octadecatrienoic acid, (Z,Z,Z)-	C_18_H_30_O_2_	278	**6.89**	2139	fatty acid
10	30.66	Octadecanoic Acid	C_18_H_36_O_2_	284	0.78	2172	sesquiterpene
11	31.52	Phytol, acetate	C_22_H_42_O_2_	338	1.65	2223	diterpene alcohol
12	36.26	Dotriacontane	C_32_H_66_	450	0.42	2302	alkanes
13	37.78	α-Sitosterol	C_29_H_50_O	414	2.38	3200	triterpene
14	38.88	α-Amyrin	C_30_H_50_O	426	**20.38**	3337	triterpene
15	39.21	Ethyl iso-allocholate	C_26_H_44_O_5_	436	2.62	3094	triterpene
16	39.91	Lupeol	C_30_H_50_O	426	**13.32**	3376	triterpene
17	40.05	Ethanol, 2-(9-octadecenyloxy)-, (Z)-	C_20_H_40_O_2_	312	**7.82**	2336	USFA
18	40.61	1-Heptacosanol	C_27_H_56_O	396	**12.15**	3016	alkane
19	42.06	Ethanol, 2-(9-Octadecenyloxy)-, (Z)-	C_20_H_40_O_2_	312	2.25	3124	SFA
20	42.34	9-Hexadecenoic acid, 9-octadecenyl ester, (Z,Z)-	C_34_H_64_O_2_	504	1.72	3584	fatty aldehyde
21	42.81	17-Pentatriacontene	C_35_H_70_	490	3.28	3508	wax
22	43.03	Vitamin E	C_29_H_50_O_2_	430	2.12	3138	aldehyde

* Unsaturated fatty acid (USFA); fatty acid methyl ester (FAME); sesquiterpene lactone (STL); saturated fatty acids (SFA).

**Table 15:** The major chemical constituents of *Salix safsafs* hexane extracts.

No.	RT	Compounds (100%)	M. F.	M. W.	Area (%)	R.I	Classification
1	13.10	1,2,3-Propanetriol, Triacetate	C_9_H_14_O_6_	218	1.64	1354	triglyceride
2	16.19	2,3-Dihydro-Benzofuran	C_8_H_8_O	120	0.21	1188	flavonoid
3	26.20	Pentadecanoic Acid, 14-Methyl-, Methyl Ester	C_17_H_34_O_2_	270	1.20	1814	FAME
4	26.94	Hexadecanoic acid	C_16_H_32_O_2_	256	3.16	1968	palmitic acid
5	29.33	9,12-Octadecadienoic Acid (Z,Z)-, Methyl Ester	C_19_H_34_O_2_	294	1.18	2093	fatty acid
6	29.47	9-Octadecenoic Acid (Z)-, Methyl Ester	C_19_H_36_O_2_	296	1.52	2095	FAME
7	37.79	α-Sitosterol	C_29_H_50_O	414	**13.86**	3200	triterpene
8	38.93	Heptacosane	C_27_H_56_	380	6.01	2700	alkane
9	40.05	Hexacosanal	C_26_H_52_O	380	**25.42**	2832	SFA
10	40.61	1-Heptacosanol	C_27_H_56_O	396	**29.51**	3016	alkane
11	42.06	Ethyl iso-allocholate	C_26_H_44_O_5_	436	10.07	3094	triterpene
12	42.81	Oleic acid, Eicosyl Ester	C_38_H_74_O_2_	562	3.82	3922	fatty acid
13	43.09	Ethanol, 2-(9-Octadecenyloxy)-, (Z)-	C_20_H_40_O_2_	312	2.40	3669	USFA

* Fatty acid methyl ester (FAME); saturated fatty alcohol (SFA); unsaturated fatty acids (USFA).

The chemical constituents of *E. camaldulensis* were identified by GC–MS analysis (Table 12, Table 13) indicating that *E. camaldulensis* contains 42 and 18 main chemical compounds in acetone and hexane extracts, respectively. 1H-Cycloprop[e]azulen-7-ol, decahydro-1,1,7-trimethyl-4-methylene-, [1ar-(1aà,4aà,7á,7aá,7bà)]- (13.64%), Ethanol, 2-(9-octadecenyloxy)-, (Z)- (8.86%), 17-Pentatriacontene (7.51%), 6-Nonenal, 3,7-dimethyl- (6.50%), 2-Cyclohexen-1-one, 4-(1-methylethyl)- (6.31%), Bicyclo(3.1.1)heptane-2,3-diol, 2,6,6-trimethyl- (4.49%), and Benzene, 1-methyl-3-(1-methylethyl)- (4.44%) for acetone extract, and Octacosanal (28.72%), Benzoic acid, 2-(2,3-dihydro-2,6-dihydroxy-7-methoxynaphtho[1,8-bc]pyran-9-yl)-5-et henyl-3-methoxy-, (R)- (12.11%), 17-Pentatriacontene (10.42%), and Betulinaldehyde (7.02%) for hexane extract (Fig. 5).

The main chemical compounds were recorded in *S. safsafs* acetone and hexane extracts were 22 and 13 phytochemical compounds, respectively. α-Amyrin (20.38%), Lupeol (13.32%),1-Heptacosanol (12.15%), n-Hexadecanoic acid (9.91%), Ethanol, 2-(9-octadecenyloxy)-, (Z)- (7.82%, 9,12,15-Octadecatrienoic acid, (Z,Z,Z)- (6.86%), and 1-Heptacosanol (29.51%), Hexacosanal (25.42%), α-Sitosterol (13.86%), Ethyl iso-allocholate (10.07%) for *S. safsafs* acetone and hexane extracts, respectively (Table 14, Table 15, Fig. 5).

### 3.6 Larvicidal field evaluation

**Fig. 6:**
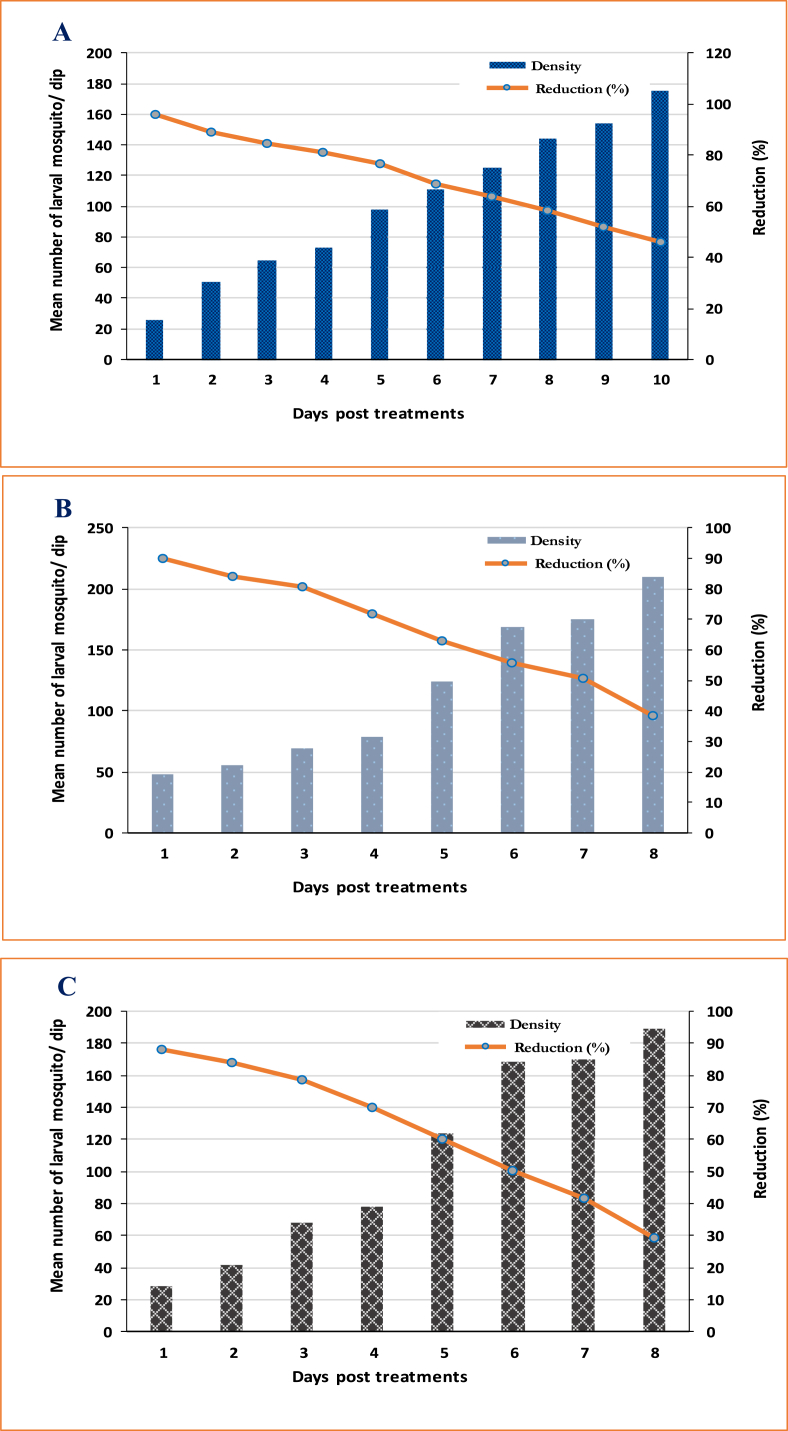
Field efficacy of *A. nilotica* (A), *E. camaldulensis* (B), and *S. safsafs* (C) treated at dose of LC_90_ X2 (529.9, 1503.5 and 902.9 ppm), respectively, in larval breeding sites.

All plant extracts lowered larval density in the small size pool at Kafr Saad village post-treatment with doses of LC_90_ X2 for *A. nilotica, E. camaldulensis, and S. safsafs* (529.9, 1503.5 and 902.9 ppm) X2, respectively). Treatments reduced larval density, where the larval reduction % of *A. nilotica* extract reached 96% at 24 h post-treatment and its persistence (>50%) reached 12 days PT, respectively (Fig. 6). The reduction percentages of *E. camaldulensis and S. safsafs* reached 90.0 and 88.2%, 24 h post-treatment, and their persistence (reduction% > 50%) reached 7 and 6 days post-treatment, respectively (Fig. 6).

## 4 Discussion

Several plants with bioactive compounds have been used with great efficacy to control and reduce a variety of medical insects and crop pests. Plants like Pyrethrum species, *Curcuma longa, Juniperus communis*, and sweet wormwood, *Artemisia annua*, are examples of plants that have been effectively used as safe pesticide sources for the management of pests and malaria vector-borne diseases [47].

Before technology, the practice of pest management using plant products was the predominant and most widely used practice, but over time, technology took hold of pesticides and synthetic pesticides were developed [48]. Due to their success in controlling typhoid fever and malaria, along with important crop diseases such as rust and blights [49,50], synthetic pesticides were more quickly accepted than plant pesticides. As a result, the use of natural products of plant origin gradually decreased until recently when the use of synthetic pesticides began to endanger public health and the environment [8,51], and monitoring of hazardous chemical pesticide residues in foods [52].

Because of this, the world has trouble with pest control and control programs, even though people keep finding new biologically active products that are widely used to fight medical, veterinary, and agricultural pests.

Our results revealed that all plant extracts in this study showed moderate to high toxicity effects against *Cx. pipiens* and *Ae. aegypti* larvae after 24 and 48 h of exposure and acetone extracts were more effective than hexane extracts.

Among the plant extracts, *A. nilotica* was the most effective regarding lethal concentrations (LC_50_) and had lower values compared to other plant extracts either in methanol or hexane extracts, at 24 and 48 h post-treatment. Parallel studies of using botanicals against *Culex* larvae were also recorded. The aqueous extract of *A. nilotica* was effectively controlled on mosquito *Culex* larvae at 2% concentration with mortality rate between 75% and 100% (LC_50_ = 400 ppm (0.004%) [53].

*Acacia nilotica* seeds and leaves have reportedly been found to have a variety of multipurpose active compounds [27,54]. As a result, extracts from the leaves and fruits of *A. nilotica* have shown promise as fungicides, bactericides, molluscicides, and insecticides [27,55].

*Acacia nilotica* seed essential oil and seed pod solvent extracts for bioefficacy against three important types of mosquitoes were evaluated by Vivekanandha565n et al. [55], 24 h post-treatment. Hydrodistilled oil from seed provided strong larvicidal activity against *Anopheles stephensi*, (LC_50_ = 5.239, LC_90_ = 9.713 mg/L); *Aedes aegypti*, (LC_50_ = 3.174, LC_90_ = 11.739 mg/L); and *Culex quinquefasciatus*, (LC_50_ = 4.112, LC_90_ = 12.325 mg/L). While the values of smoke toxicities were 82% in *Cx. quinquefasciatus*, 90% in *Ae. aegypti*, and 80% mortality in *An. stephensi* adults [55].

Our data agreed with the work of Zaitoun et al. [56], who studied the effect of *A. nilotica* extracts on the larval *Cx. pipiens*. They found that *A. nilotica* acetone leaf extracts were acutely toxic at 212.1 mg/L and chronically toxic at 144.2 mg/L; in addition, acetone and hexane extracts have demonstrated the capacity to inhibit egg hatchability and adult emergence.

The extracts of the *Acacia* plant are characterized by various phytochemicals, including terpenes, unsaturated and monounsaturated fatty acids, carboxylic acid, alkanes, and other plant extracts. Terpenoids, terpenes, and carbonyl were found to be the most effective phytochemical insecticides when tested against beetles, mosquitoes, caterpillars larvae and other flies over a 76-year period of literature review and meta-analyses of phytochemical insecticides [57].

Squalene is a multipotent triterpenoid widely present in a variety of plants, as in Acacia nilotica. Many studies have reported the pharmacological efficacies of squalene, including its antioxidant, antimicrobial, anticarcinogenic, and bioinsecticide properties [58,59].

The use of plant secondary metabolites and essential oils against mosquito larvae and adults has been documented in several laboratory studies [60,61]. *Acacia nilotica* crude extracts have been shown to be toxic to larvae of many common mosquito species [27,56].

Researchers have found that the *Acacia nilotica* plant has effects against HCV and HIV–I having are antioxidant, anti-malarial, anti-cancer, anti-plasmodial, anti-molluscicidal, anti-fungal, anti-microbial, and anti-plasmodial activities [14,62].

Besides the efficacy of *A. nilotica* on mosquito larvae, the methanol and acetone bark extracts of *A. nilotica* were tested for decreased growth of 1st, 2nd, and 3rd instar *Bactrocera cucurbitae* larvae using an artificial diet bioassay [63]. The authors showed that both extracts had a negative impact on *B. cucurbitae*, a dangerous pest of cucurbit crops, during both their larval stage and overall developmental span. While significantly inhibited pupation and emergence percentages were recorded [63].

The literature review reveals that there have been very few investigations on the effects of willow leaf extracts on mosquito larvae. A study by Sameeh et al. [64] showed that the LC_50_ value of an ethanol extract of *Salix willow* leaves against *An. pharoensis* larvae were 73.1 ppm, which is noticeable when combined with the 24 h (LC_50_ = 2100 ppm) against 4th instar larvae of *Cx. pipiens* [65].

*Salix safsaf*, the deciduous herb, cape silver willow, or safsaf willow, is another name for the little tree known as the Egyptian willow, which has been found in Egypt since prehistoric times [38,39]. Typically, it can be found in moist places like those near waterways and on the Nile River in Egypt. Salicin willow, another name for white willow, has long been used for its therapeutic properties [40]. Its branches are supple, long, and thin. Over many generations, the plant's leaves, seeds, and other parts have been used in traditional medicine to relieve inflammation, pain, and fever [41].

Besides the evaluation of extracts of the willow tree (*Salix safsaf*) on mosquitoes is very rare, in addition to extracting the compound acetylsalicylic acid (Aspirin) from willow tree [66], which has multiple medical uses [67].

13 plant extracts were evaluated against *Musca domestica*. The prickly pear (*Opuntia vulgaris*) and sugarcane tree (*Saccharum* spp.) were excluded from the preliminary toxicity against *M. domestica* adult at 300 and 1000 ppm because they displayed very low toxicity even at the higher dose. According to their effectiveness, the bioassayed extracts could be grouped as follows: *Salix safsaf* (0.24 mg/cm^2^), *Conyza aegyptiaca* (0.25 mg/cm^2^), *Azadirachta indica* (0.28 mg/cm^2^) is followed by five extracts with the same RC_50_ value (Recovery concentration) was 0.29 mg/cm^2^ and (*Sonchus oleracues, Citrus aurantifolia, Cichorium intybus, Zea mays* and *Piper nigrum*) [68].

*Salix safsaf* leaf extracts were investigated for insecticidal efficacy, growth regulation, adult performance, and repellency against third-instar larvae of the housefly *Musca domestica* by Hasaballah et al. [69]. Data obtained showed that the percentage of deaths among pupae and third-instar larvae rose with concentration, where the LC_50_ values were 467.714 and 793.348 ppm in petroleum ether, methanol (423.22 and 710.18 ppm), chloroform (533.55 and 942.88 ppm), and ethanol (384.90 and 712.33 ppm).

Previous studies on the genus Salix's phytochemical composition have produced findings on phenolic substances, flavonoids, terpenes, and lignans [70,71]. Salicylic glycosides, the most prevalent phenolic compounds found, have been shown to have analgesic, antipyretic, anti-inflammatory, and anti-rheumatic, antioxidant and anticancer activities [71,72].

Our data showed that the GC-MS analysis for *A. nilotica* extracts indicated the presence of 2,3-di-n-nonylanthraene (13.69%), 1,2,3-benzenetriol (13.46%), 9,12,15-Octadecatrienoic acid, (Z,Z,Z)- (11.16%), Squalene (8.79%), n-Hexadecanoic acid (6.86%), and 9-Octadecenoic acid, methyl ester, (E)- (6.40%) for acetone extract, and dl-à-Tocopherol (16.88%), Squalene (14.74), 17-Pentatriacontene (10.58%), Stigmasterol (9.75%), and 9,12,15-Octadecatrienoic acid, (Z,Z,Z)- (7.46) for *A. nilotica* hexane. The main compounds detected were phenolics or alkaline pyrogallol, fatty acid, sesquiterpene, triterpene, fatty acid methyl ester and palmitic Acid (waxes), which are the main and active compounds, and which may be involved in insecticidal activity [[73], [74], [75]]. Besides, biochemical analysis using GC-MS chromatogram showed that the *A. nilotica* plant contains a photosensitizer (Benz [E] Acephenanthrylen-12-ol) (1.03%), and this distinguishes the amber color of *A. nilotica* extracts that are with the passage of time it turns to dark brown and then black.

The chemical constituents of *E. camaldulensis* were identified by GC–MS analysis indicating that *E. camaldulensis* contains 1H-Cycloprop[e]azulen-7-ol, decahydro-1,1,7-trimethyl-4-methylene-, [1ar-(1aà,4aà,7á,7aá,7bà)]- (13.64), Ethanol, 2-(9-octadecenyloxy)-, (Z)- (8.86%), 17-Pentatriacontene (7.51), 6-Nonenal, 3,7-dimethyl- (6.50%), and 2-Cyclohexen-1-one, 4-(1-methylethyl)- (6.31%) for acetone extract, and Octacosanal (28.72%), Benzoic acid, 2-(2,3-dihydro-2,6-dihydroxy-7-methoxynaphtho [1,8-bc]pyran-9-yl)-5-ethenyl-3-methoxy-, (R)- (12.11%), and 17-Pentatriacontene (10.42%), for hexane extract and all these compounds are sesquiterpene, unsaturated fatty acids, and phenols.

In addition to the GC–MS profile of the *S. safsafs* acetone and hexane extracts, which showed that willow contain main chemical compounds were α-Amyrin (20.38%), and Lupeol (13.32%) that are triterpene, and 1-Heptacosanol (12.15%) and n-Hexadecanoic acid (9.91%) are fatty acids in *S. safsafs* acetone extracts. 1-Heptacosanol (29.51%) is an alkane and Hexacosanal (25.42%) is saturated fatty alcohol, while α-Sitosterol (13.86%) and Ethyl iso-allocholate (10.07%) are triterpene for *S. safsafs* hexane extracts, respectively, which are considered essential compounds.

Secondary metabolites are a diverse class of molecules that plants manufacture to protect them from herbivores and microbes. Alkaloids, phenolics, and terpenoids make up a large portion of these secondary metabolites and these compounds are toxic substances against insects [76].

Pharmacological studies are closely related to phytochemical compounds. The goal of these studies is to find out how to use many of these phytochemical compounds for different medical, therapeutic, and cosmetic purposes. These phytochemicals, derived or extracted from several aromatic plants such as *Malva sylvestris, A. nilotica, Ageratina pichinchensis, E. camaldulensis, Capparis spinosa*, and many other aromatic plants, are responsible for many pharmacological activities such as anti-inflammatory, antimicrobial, hepatoprotective, laxative, antiproliferative, and antioxidant [[77], [78], [79], [80], [81]].

Similar studies indicated that methanol leave extract of *A. nilotica* contained calycanthidine, linoleic acid, catechine, malic acid and octadeconic acid [82]. The GC-MS profile of seed essential oil from *A. nilotica* showed the presence of hexadecane (18.440%) and heptacosane (15.914%), which are the main and active compounds, and which may be involved in insecticidal activity [83].

Using GC/MS analyses for determine the phytochemistry and larvicidal activity of *Eucalyptus camaldulensis* against *Anopheles stephensi*, it was discovered that 28 components, or 99.60% of the total oil, were present. 1,8-cineole (69.46%), γ-terpinene (15.10%), α-pinene (5.47%) and globulol (2%), were the primary components of the leaf essential oil. The larvicidal activity of the leaf extract and volatile oil was substantial, with LC50 values of 89.85 and 397.75 ppm, respectively [36].

The antioxidant activity of nonvolatile chemicals in leaves of *Eucalyptus camaldulensis* trees was investigated, where the extracts obtained by ethanol digestion and supercritical fluid extraction (SFE; CO_2_ with 15% ethanol) showed a new promising antioxidative activities [84].

*Eucalyptus camaldulensis* leaves extracts showed the gallic and ellagic acid were found to be the prevailing antioxidants in the ethanolic extract and the main two compounds; 5-hydroxy-7,49-dimethoxy flavone and 5-hydroxy-7,49-dimethoxy-8-methyl flavone of the supercritical fluid extraction extract with antioxidative activity revealed to be flavones [84,85].

The fruit volatile oil of *E. camaldulensis* var. *brevirostris* was extracted by hydrodistillation and ethanol, where the gas chromatography-mass spectrometry was used to analyses the volatile oil that was produced. There were 38 volatile components found. Aromadendrene (17.99%), α-pinene (12.68%), cubenol (9.23%), α-gurjunene (6.65%), *p*-cymenene (5.39%), thymol (1.62%), and p-cymen-7-ol (0.73%) were the primary volatile components in the fruit volatile oil. Monoterpenes (20.6%), sesquiterpenes (33.8%), light-oxygenated (8.1%), and heavily oxygenated (37.6%) compounds were the four groups into which the volatile components were divided [86].

Our data showed that the extracts of *A. nilotica* are characterized by an amber color, and with the passing of time, they change to a dark brown color and then black, so the plant extracts were evaluated against mosquito larvae, *Cx. pipiens* and *Ae. aegypti* at diluted concentrations (62.5 and 125 ppm) in sunlight and shadow light. The biochemical analysis with the GC-MS chromatogram confirmed that the *A. nilotica* extracts contains a photosensitizer (Benz[e]acephenanthrylen-12-ol), which explains the highly toxic effects on mosquito larvae, where the mortality rates in sunlight exposure reached 93% and 80% at acetone and hexane extracts against *Cx. pipiens,* 125 ppm, respectively. While in the shadow, the mortality rates % reached 34, and 21%, respectively. Similarly, in sunlight, the mortality rates were 79% and 70% in acetone and hexane extracts, respectively against *Ae. aegypti* larvae, while in shadow light, the mortality rates were 25% and 18%, respectively.

According to reports, many medicinal plants may contain various photosensitizer components with anti-tumor potential and other treatments. Plants containing chemical components that have a photosensitizing effect include, *Scutellaria barbata, Heterophyllaea pustulata, Chelidonium majus, Echinops latifolius, Annona purpurea, Helleborus niger,* and *Aglaonema simplex*. Natural substances with photodynamic action with various cellular targets include, Phthalocyanine, Hypocrellin A, Tolyporphin, Carvacrol, Hypericin, etc. [87,88].

The data of the present work confirmed that the extract of A. nilotica contains naturally occurring photosensitizing substances such as Fluoromethacin, dl-α-Tocopherol and Benz[E]acephenanthrylen-12-OL.

Natural photosensitizing compounds are considered safe mechanisms in the control of insect pests, in addition to their use in photodynamic therapy, which is a minimally invasive, alternative, and promising treatment for several diseases, including cancer, actinic keratosis, atherosclerotic plaques, and macular degeneration and other diseases [88].

Insects have a significant negative impact on agriculture all over the world other than transmitting many deadly diseases to humans or animals. Crops have created defenses against these pests, including a variety of specific compounds based on secondary metabolites. These substances (phytoalexins, phytocytibins) can either directly affect pests or can be used unintentionally to attract their natural enemies [89].

## 5 Conclusions

The diseases that mosquitoes spread around the world must be prevented from fatally affecting humans and their animals. The basis of pest control is the use of traditional pesticides, yet virtually all pesticides are no longer effective against insects. Due to the great variety and high efficacy of phytochemicals compounds, the use of natural products as ecologically benign pesticides is currently a significant topic of research. Therefore, this study revealed for the first time the efficacy of acetone and hexane extracts of *A. nilotica, E. camaldulensis,* and *S. safsafs* against larvae and pupae of *Culex pipiens* and *Aedes aegypti*. Acetone plant extracts were more recommended than hexane extracts as an ideal eco-friendly and inexpensive pest control approach that could be incorporated into integrated pest management used for protecting human from vector-borne diseases. All plant extracts in this study showed moderate to high toxic effects against *Cx. pipiens* and *Ae. aegypti*, beside that *A. nilotica* was the most effective regarding lethal concentration, while willow extracts showed a clear effect on the delay of larval development. A. nilotica had a higher mortality rate in sunlight than in shade, either from acetone or hexane extracts because they contained the photosensitizer. *A. nilotica* had a higher mortality rate in sunlight than in shadow light, either from acetone or hexane extracts because they contained the photosensitizer (Benz [E] cephenanthrylen-12-ol) (1.03%), and this distinguishes the amber color of *A. nilotica* extracts that are with the passage of time it turns to dark brown and then black. General, local, regional, and rural populations with few other control options can manage mosquito vectors by using such eco-friendly, inexpensive plant extracts in safe phytochemical pesticides. Further studies could be directed towards studying the phytochemical compounds and field application, and the safety profile of *Acacia nilotica, Eucalyptus camaldulensis,* and *Salix safsafs* against non-target organisms.

## Author contribution statement

Rowida S. Baeshen: Conceived and designed the experiments; Analyzed and interpreted the data; Contributed reagents, materials, analysis tools or data; Wrote the paper.

Mohamed M. Baz: Conceived and designed the experiments; Performed the experiments; Analyzed and interpreted the data; Contributed reagents, materials, analysis tools or data; Wrote the paper.

## Data availability statement

Data will be made available on request.

Funding**:** This research received no external funding

Institutional Review Board Statement: Not applicable.

Informed Consent Statement: Not applicable.

## Declaration of competing interest

The authors declare that they have no known competing financial interests or personal relationships that could have appeared to influence the work reported in this paper.
